# Genome-wide identification and association analysis of informative SNPs of various nutri-nutraceutical traits in Buckwheat (*Fagopyrum* spp.)

**DOI:** 10.3389/fpls.2025.1559621

**Published:** 2025-04-24

**Authors:** Madhiya Manzoor, Jebi Sudan, Adil Nath, Basharat Bhat, Parvaze A. Sofi, M. Ashraf Bhat, P. V. Vara Prasad, Sajad Majeed Zargar

**Affiliations:** ^1^ Proteomics Lab, Division of Plant Biotechnology, Sher-e-Kashmir University of Agricultural Sciences and Technology of Kashmir, Srinagar, Jammu & Kashmir, India; ^2^ Center of Artificial Intelligence and Machine Learning (CAIML), Sher-e-Kashmir University of Agricultural Sciences and Technology of Kashmir, Srinagar, Jammu & Kashmir, India; ^3^ Division of Genetics and Plant Breeding, Sher-e-Kashmir University of Agricultural Sciences and Technology of Kashmir, Srinagar, Jammu & Kashmir, India; ^4^ Sustainable Intensification Innovation Lab, Kansas State University, Manhattan, KS, United States

**Keywords:** buckwheat, nutraceutical, single nucleotide polymorphisms, genotyping by sequencing, genome wide association studies, quantitative trait loci

## Abstract

Buckwheat (*Fagopyrum* spp.) is a pseudocereal with nutraceutical properties that offer several nutritional and health benefits. Buckwheat proteins are gluten-free and have balanced quantities of amino acids and micronutrients, with a higher content of health-promoting bioactive flavonoids that make it a golden crop of the future. In the present study, we conducted a genome-wide association study (GWAS) to investigate the genetic basis of nutraceutical traits in buckwheat. Using 132 diverse genotypes, we evaluated 10 key nutritional and nutraceutical traits: phenol, flavonoids, antioxidants, methionine, lysine, protein content, nitrogen, iron, zinc, and ascorbic acid. *Fagopyrum tartaricum* displayed higher levels of phenols, flavonoids, antioxidants, iron, zinc, and nitrogen, while *Fagopyrum esculentum* exhibited elevated methionine, lysine, protein, and ascorbic acid levels. Genotyping by sequencing identified 3,728,028 single-nucleotide polymorphisms (SNPs), with the highest density on chromosome 1. GWAS detected 46 significant SNPs associated with the studied traits, including an SNP on chromosome 6 linked to lysine with aphenotypic contribution of 49.62%. Candidate gene analysis identified 138 genes within 100 kb of significant quantitative trait loci (QTLs), involved in metabolic and biosynthetic pathways such as amino acid and carbohydrate metabolism. Population structure analysis grouped the genotypes into three populations, enhancing the reliability of marker-trait associations. Gene Ontology analysis highlighted key biological processes, including lipid transport, tryptophan metabolism, and protein phosphorylation, providing insights into the molecular mechanisms governing these traits. The present study emphasizes the potential of molecular breeding to enhance the nutritional quality of buckwheat and its role in addressing global malnutrition. The identified SNP markers and candidate genes offer a valuable foundation for developing high-yield, nutrient-rich buckwheat varieties through genome editing and marker-assisted selection.

## 1 Introduction

Buckwheat (*Fagopyrum* spp.) is one of the most important pseudocereals that grow in hilly areas, especially in the Himalayas above 1,500 to 4,500 m sea level. It is considered as the poor man’s crop and is an alternate cereal that represents an important food supply in remote places in the Himalayas. Out of nearly 30 species belonging to the genus *Fagopyrum*, only two species, viz., *Fagopyrum esculentum* (common buckwheat) and *Fagopyrum tataricum* (tartary buckwheat), are cultivated. They are diploid (2*n* = 2*x* = 16) (Farooq et al., 2016) with an estimated genome size of 505 Mb for *F. tataricum* and 1,340 Mb for *F. esculentum*. Buckwheat as a nutraceutical crop has been recently introduced to many countries in the form of flour and groats, and the food prepared from it have beneficial effects on human health (Nepali et al., 2019). The nutritional and nutraceutical characteristic is due to the high concentration of essential amino acids, micronutrients (Fe and Zn), antioxidants, phenols, flavonoids, ascorbic acid, folate, resistant starch, and fiber (Bonafaccia and Fabjan, 2023; Manzoor et al., 2023; Sofi et al., 2023). It is also a good source of bioactive flavonoids such as rutin, quercetin, (iso)vitexin, and epicatechin, which have proven beneficial effects on diabetes, hypertension, and hyperlipidemia (Joshi et al., 2020; Zargar et al., 2023a). Buckwheat is gluten-free, making it an excellent choice for individuals with celiac disease or gluten sensitivity. Buckwheat contains all nine essential amino acids, including lysine, which is often lacking in other grains. Its high antioxidant content, particularly rutin, supports heart health by improving blood circulation and reducing inflammation. Additionally, its low glycemic index helps regulate blood sugar levels, benefiting diabetics. Buckwheat promotes digestive health due to its high fiber content and is a valuable food for maintaining overall wellbeing and balanced nutrition.

Nutritional security is a major concern in the present era and breeding for a nutrient-dense crop such as buckwheat will provide a sustainable solution. Buckwheat, despite its high economic, agricultural, and nutritional significance, is considered a minor or underutilized crop. With the declining production and productivity, improvement in the nutritional aspect is challenging for this crop. The lack of adequate intra-specific polymorphisms in buckwheat has hindered the identification of robust genes or quantitative trait loci (QTLs) linked to various nutritional and nutraceutical traits for marker-assisted genetic improvement. To address this issue, it is essential to utilize diverse buckwheat germplasm for mapping desirable QTLs and then use high-throughput genotyping platforms to generate genotypic data for the discovery of genome-wide markers for multiple traits. This approach is crucial for dissecting complex quantitative traits in buckwheat and for the subsequent map-based QTL cloning. Although various genetic molecular marker systems have been developed in buckwheat (Konishi et al., 2006; Hara et al., 2011; Yabe et al., 2014; Yasui et al., 2016), the use of single-nucleotide polymorphism (SNP) markers in buckwheat remains limited.

SNPs are considered the most efficient markers for genome-wide association studies (GWASs) because of their abundant nature, heritability, and high resolution. GWAS, also known as association mapping or linkage disequilibrium (LD) mapping study, is preferred as it takes full advantage of the high phenotypic variation within a species and the high number of historical recombination events in the natural population. To identify the genetic loci underlying traits at a relatively high resolution, GWAS has become an alternative approach to the conventional QTL mapping (Wei, 2016). In a number of economically valuable crops, GWAS has been used to gain insight into the genetic architecture of important nutritional traits; however, no such efforts have been made in terms of exceptionally nutritional and nutraceutical rich buckwheat. Recently, we have used the genotyping by sequencing (GBS) approach for large-scale SNP marker mining and genome-wide association analysis for various metabolites and yield-attributing traits in buckwheat (Zargar et al., 2023b; Naik et al., 2024).

In the current study, we integrate the high-throughput genotyping data with various nutritional and nutraceuticals traits for the identification of stable QTLs and their respective candidate genes.

## 2 Material and methods

### 2.1 Plant material

In the present study, a diverse set of 132 buckwheat genotypes was used that includes the germplasm collected from different regions of the Northwestern Himalayas along with some accessions procured from NBPGR, New Delhi. The details of all 132 genotypes collected are given in **Supplementary Table 1**. The material comprised both common (*F. esculentum*) and tartary (*F. tartaricum*) buckwheat germplasm. The core set represented a highly variable material with respect to morphological, phenological, and yield traits as reported in our earlier studies, with wide ranges reported for economically important traits. The collected germplasm was purified and cultivated at the experimental field of Sher-e-Kashmir University of Agricultural Sciences and Technology of Kashmir (latitude 35°C30′N, longitude 75°C15′E, and altitude 1,700 m) during 2020–2021 and 2021–2022. The harvested plant materials (seeds) were used for biochemical analysis after preparing flour from each genotype. In order to reduce the error and increase the precision of the experiment, three biological replicates of each genotype for every biochemical (nutritional/nutraceutical) parameter was considered.

### 2.2 Nutritional and nutraceutical profiling

The buckwheat seed material of each genotype was powdered to analyze traits, which include phenol, flavonoid, antioxidants, methionine, lysine, protein content, nitrogen, iron, zinc, and ascorbic acid.

#### 2.2.1 Total phenols, flavonoids, and antioxidant profiling

Total phenol content was estimated via the spectrophotometric method using Folin Ciocalteu’s Reagent (FCR) (Samatha et al., 2012). Phenols react with the oxidizing agent phosphomolybdate in FCR to generate molybdenum blue, a blue-colored compound at a wavelength of 725 nm. Total flavonoid content was estimated by the aluminium chloride method. Flavonoids develop a brick red color with AlCl_3_ and NaNO_2_ at alkaline pH (Talari et al., 2012). The absorbance of the complex was recorded at 510 nm. The DPPH (2,2-diphenyl-1-picrylhydrazyl) assay (Brand-Williams et al., 1995) was carried out to assess the antioxidant property of the plant extracts.

#### 2.2.2 Amino acid profiling

For lysine estimation, 100 mg of finely ground sample was taken, to which 5 ml of papain was added and incubated overnight at 65°CC. Then, it was cooled to room temperature and centrifuged for 5 min, and the supernatant was collected. One milliliter of solution was taken out from each tube and 0.5 ml each of amino acid mixture and copper phosphate suspension was added. This mixture was vortexed for 5 min followed by centrifugation. 2-Chloro-3,5 dinitropyridine solution (0.1 ml) was added to 1 mL of supernatant and was mixed well and shaken for 2 h. HCl (1.2 N) was added and mixed. Extraction was done by 5 mL of ethyl acetate and the top layer was discarded. Absorbance of aqueous layer at 390 nm was measured with blank (5 mL of papain) (Sandhya et al., 2020).

For methionine estimation, 500 mg of sample was autoclaved with 6 mL of 2 N HCl for 1 h. The sample was heated to boil with a pinch of charcoal and filtered through Whatman filter paper. The pH of the filtrate was adjusted to 6.5 with 10 N NaOH and volume was raised to 50 mL and then it was transferred to a conical flask. Three milliliters of 10% NaOH and 0.15 mL of 10% sodium nitroprusside were added. Absorbance of red color was read at 520 nm in a spectrophotometer (Thakur and Guttikonda, 2017).

#### 2.2.3 Seed nitrogen and protein content

The total nitrogen is estimated by micro-Kjeldahl method (Barbano et al., 1990). In this method, 2 g of sieved sample was taken in the Kjeldahl cylindrical tube and 25 mL of 0.32% potassium permanganate and 20 mL of 2% boric acid in a conical flask were added and both the cylindrical tube and the conical flask were placed in the Kjeldahl assembly. Thirty milliliters of 2.5% sodium hydroxide was added into the cylindrical tube, and the heat distils out ammonium gas that was collected in the conical flask. The contents were titrated in conical flask against 0.02 N sulfuric acid till color changed from green to pink. Similarly, blank titration was carried out.

Total protein content was calculated by the following formula:


Total Protein Content(%)=Nitrogen content(%)×6.25

#### 2.2.4 Fe, Zn, and ascorbic acid estimation

For the estimation of Fe and Zn, 0.5 g of buckwheat seeds from each sample was taken for di-acid digestion. To this, 20 mL of the digestion solution (a mixture of nitric acid and perchloric acid at a ratio of 9:4 V/V) was added. The mixture was left at room temperature overnight, and the next day, this mixture was kept on a hot plate and heated slowly until a clear solution was obtained and brownish smoke is no longer released, indicating complete digestion of the organic matter. This clear solution was allowed to cool at room temperature, and the digested sample was transferred to 50-mL volumetric flasks. The transfer was done by using ash-free quantitative filter paper (No. 1). The volume of the solutions was raised to 50 mL using distilled water. The samples were then further diluted, and the concentration of these minerals was determined by using ICP-OES (inductively coupled plasma optical emission spectrometry) (Karpiuk et al., 2016).

For ascorbic acid estimation, 0.5 g of buckwheat seed powder was taken and dissolved in 20 mL of 4% oxalic acid solution. Then, the buckwheat solution was filtered and liquid was collected. Then an aliquot of 10 mL was transferred to a conical flask and bromine water was added dropwise with constant mixing and then 2 mL of extract was pipetted out and volume was made up to 3 mL by adding distilled water. Then, 1 mL of DNPH reagent was added followed by two drops of thiourea in each tube. The contents in the tube were mixed thoroughly and were incubated at 37°C for 3 h. After incubation, 7 mL of 80% sulfuric acid was added and absorbance was measured at 540 nm (Pisoschi et al., 2011).

### 2.3 Statistical analysis

All the biochemical observations were recorded in three replicates and values were then averaged. One-way analysis of variance (ANOVA) was applied to evaluate the variance of these traits among the genotypes and Pearson’s pairwise correlation coefficient was calculated for all traits using the R² programme and heatmap was generated among all the traits. Frequency distribution analysis was also done for all the traits of buckwheat.

### 2.4 Library preparation and genotyping by sequencing

Seeds of 132 genotypes of buckwheat were sown in plastic trays for 3 weeks in a polyhouse and the harvested shoots were used for genomic DNA extraction using the CTAB method (Doyle, 1991), and the quality and quantity of DNA were checked on both gel electrophoresis and nano-drop (mySPEC, Wilmington, USA). GBS libraries were prepared by the already standardized method (Elshire et al., 2011) with little modification. DNA (100 ng) was digested for 4 h at 75°C with ApeKI (New England Biolabs, Ipswitch, MA) in 20 µL volumes containing 1× NEB Buffer and 3.6 U ApeKI. Barcoded adapters were then ligated to sticky ends by adding 30 µL of a solution containing 1.66× ligase buffer with ATP and T4 ligase (New England Biolabs) to each well. Samples were incubated at 22°C for 1 h and heated to 65°C for 30 min to inactivate the T4 ligase. Sets of 132 digested DNA samples, each with a different barcode adapter, were combined (5 µL each) and purified using a commercial kit (QIA quick PCR Purification Kit; Qiagen, Valencia, CA) according to the manufacturer’s instructions. DNA samples were eluted to a final volume of 25 µL. Restriction fragments from each library were then amplified in 50-µL volumes containing 10 µL of pooled DNA fragments, 25 µL of KAPA HiFi Hot Start Ready Mix PCR, and 1 µL each of the P5 and P7 dual indexing primers (12.5 pmol). These primers contained complementary sequences for amplifying restriction fragments with ligated adapters, binding PCR products to oligonucleotides that coat the Illumina sequencing flow cell and priming subsequent DNA sequencing reactions. The final PCR products were purified with 0.9× AMPure XP beads (catalog: A63881, Beckman Coulter) to remove unused primers. The purified 132-plex final DNA library was quantified using a Bioanalyzer (Agilent Technologies) and sequenced on a single lane of Illumina HiSeq X10 platform (Illumina Inc., San Diego, CA, USA) using V4 sequencing chemistry.

### 2.5 Post sequencing analysis

The raw reads were filtered for adapter sequences, low-quality reads, and low-quality residues towards the 5′ region of the sequence. After quality filtering and data de-multiplexing, the high-quality sequences were mapped to the Tartary buckwheat reference genome assembly (GCA_002319775.1, URL: https://www.ncbi.nlm.nih.gov/datasets/genome/GCA_002319775.1/) using BWA program V 0.7.5 (Li et al., 2009). SNPs were mined from the coding and non-coding regions and were subsequently annotated. The SNPs were annotated to the genic, intergenic, non-coding, and regulatory regions using the SNPEFF program (Cingolani et al., 2012; De Pablo et al., 2012). Moreover, a comprehensive comparison of the genetic sequences at the genomic level between *F. esculentum* (Common Buckwheat) and *F. tartaricum* (Tartary Buckwheat) was performed through pairwise genome alignment, using GSALIGN program (https://github.com/hsinnan75/GSAlign). This process aimed to elucidate the shared characteristics and distinctions within the genomes of these two buckwheat species. The pairwise genome alignment between the two buckwheat genomes encompassed a series of steps, ensuring accurate and reliable results. Initially, the genomic data of both species underwent a preprocessing stage to eliminate any extraneous elements that might introduce noise and potentially hinder the alignment process. By reducing unwanted artifacts, the subsequent alignment was enhanced, allowing for more precise comparison of the genetic sequences. To optimize the alignment, GSALIGN tends to maximize the similarity between corresponding regions while minimizing any gaps that might occur in the alignment. By strategically aligning the sequences, the software facilitated the identification and comparison of specific genetic elements shared between the two species. The results were visualized using DotPlot (https://dotplot.soft112.com/).

### 2.6 Population structure analysis

Population structure was estimated using a Bayesian Markov Chain Monte Carlo model (MCMC) implemented in STRUCTURE v2.3.4 (Pritchard et al., 2000). A total of 944 filtered SNPs were converted to structure format using PGD Spider version 2.1.1.5. Three runs were performed for each number of population (*K*) set from 2 to 7. Burn-in time and MCMC replication number were set to 100,000 and 300,000, respectively, for each run. The most probable *K*-value was determined by Structure Harvester, using the log probability of the data [Ln*P*(*D*)] and delta *K* (Δ*K*) based on the rate of change in [Ln*P*(*D*)] between successive populations. The neighbor-joining tree was built using Phylip and MEGA5 (Felsenstein, 1989; Tamura et al., 2011).

### 2.7 Principal component analysis

Principal component analysis (PCA) was calculated using PLINKV 1.9 (Purcell et al., 2007) and then plotted by using the R program. Dendrogram analysis was done using TASSEL V4 using the neighbor-joining method and then plotted with Structure Q-matrix using iTOL. A PCA plot was made on four populations that were detected using Structure. The population structure was scored for K-values ranging from 1 to 12 across the panel using high-quality SNPs.

### 2.8 Marker–trait association

A compressed mixed linear model (CMLM) was used using GAPIT V3 (Lipka et al., 2012), which is an R package that performs a GWAS and genome prediction (or selection). This program uses state-of-the-art methods developed for statistical genetics, such as the unified mixed model, EMMA, CMLM, and P3D/EMMAx. SNPs were considered significant using threshold log10 (*p*-value) < 1e−4. A threshold of *p* < 1e−4 balances sensitivity (the ability to detect true associations) and specificity (the ability to avoid false positives). It is stringent enough to minimize false positives while still capturing potentially meaningful associations. Moreover, using a threshold of *p* < 1e−4 allows researchers to focus on associations with stronger statistical support, making subsequent analysis more manageable and interpretable. Manhattan plots and quantile–quantile (QQ) plots were developed using the R-package QQMAN. Manhattan plots demonstrated statistically significant associated markers, and QQ plots were prepared to graphically visualize the distribution pattern for associated markers. The *r* ^2^ values for markers were calculated using GAPIT. There are typically two threshold lines to distinguish statistically significant associations. The higher threshold line represents the associations that are considered significant after correcting for multiple testing across the entire genome. Points above this line indicate associations that are highly likely to be true positives and are of particular interest; meanwhile, the lower threshold line serves as a reference point for associations that may not meet the genome-wide significance threshold but still show potential for being meaningful.

### 2.9 LD plot

LD was measured by the parameter *r* ^2^ using SNPs with high confidence. The values were calculated using TASSEL v5.0 (Bradbury et al., 2008), and the values were plotted against genetic distance (in bp) in R software. A threshold of *r* ^2^ = 0.2 was used to determine LD extent. The *r* ^2^ values were plotted against the physical distance among markers, and the second LOESS decay curve was fitted to determine the size of LD blocks.

### 2.10 Candidate gene identification and Gene Ontology annotation

The gene containing the SNP was used to determine the probable candidate gene search from the significant SNP–trait associations obtained from GWAS using the SNPEFF program V5.1 against *F. esculentum* annotation downloaded from NCBI (Cingolani et al., 2012). The candidate genes were mapped to the Kyoto Encyclopaedia of Genes and Genomes (KEGG) database using the KEGG-KAAS (KEGG Automatic Annotation Server) server for pathway analysis and Gene Ontology (GO) annotation was carried out using stand-alone BLASTP and BLASTX (Johnson, 2008) against the UniProt database (release 2022_02) to gain insight into the functional role of candidate genes with SNPs (The UniProt Consortium, 2019).

## 3 Results

### 3.1 Nutritional and nutraceutical profiling

**Figure 1:**
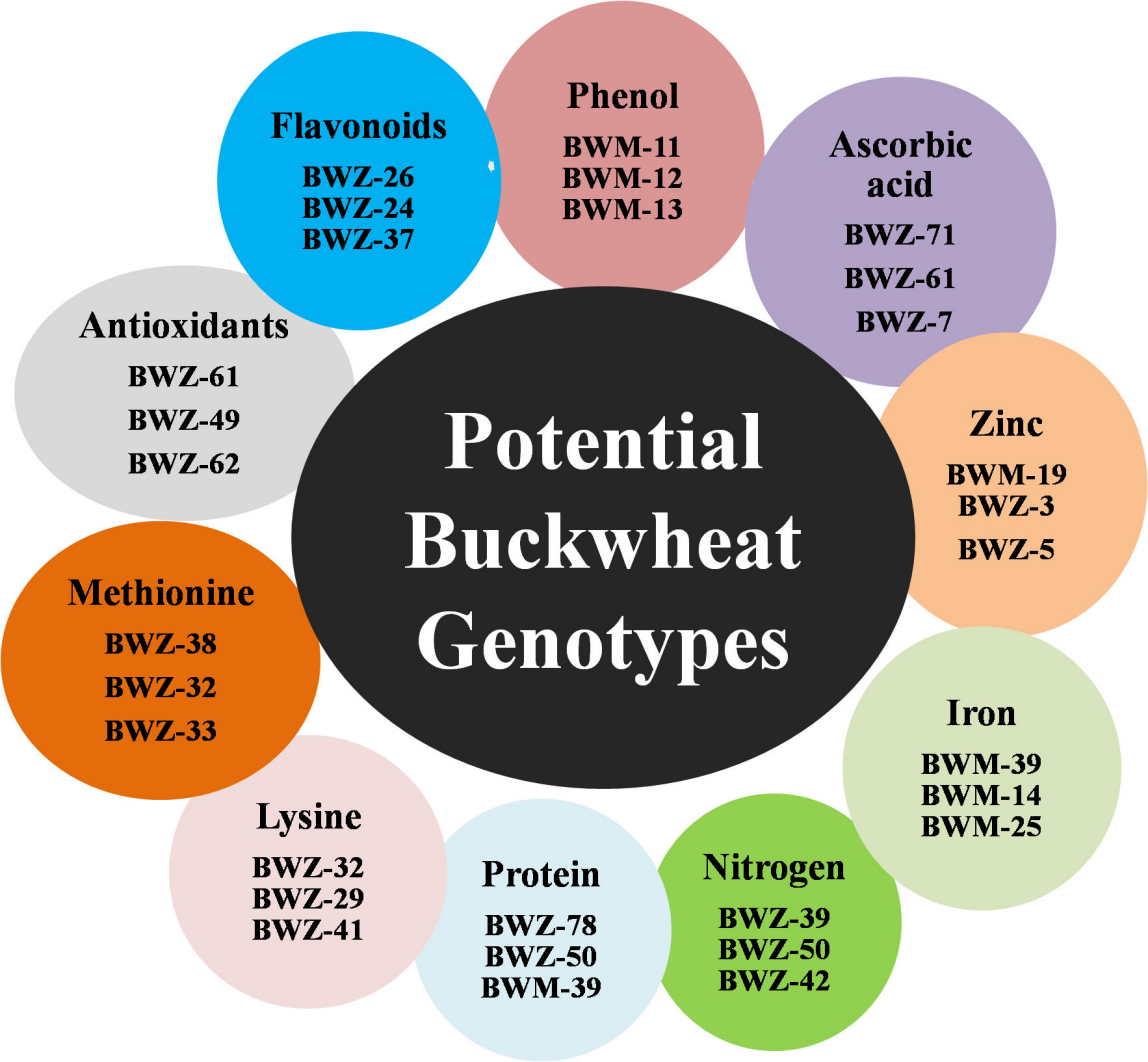
The list of buckwheat genotypes with high concentration of different nutri-nutraceutical contents.

**Figure 2:**
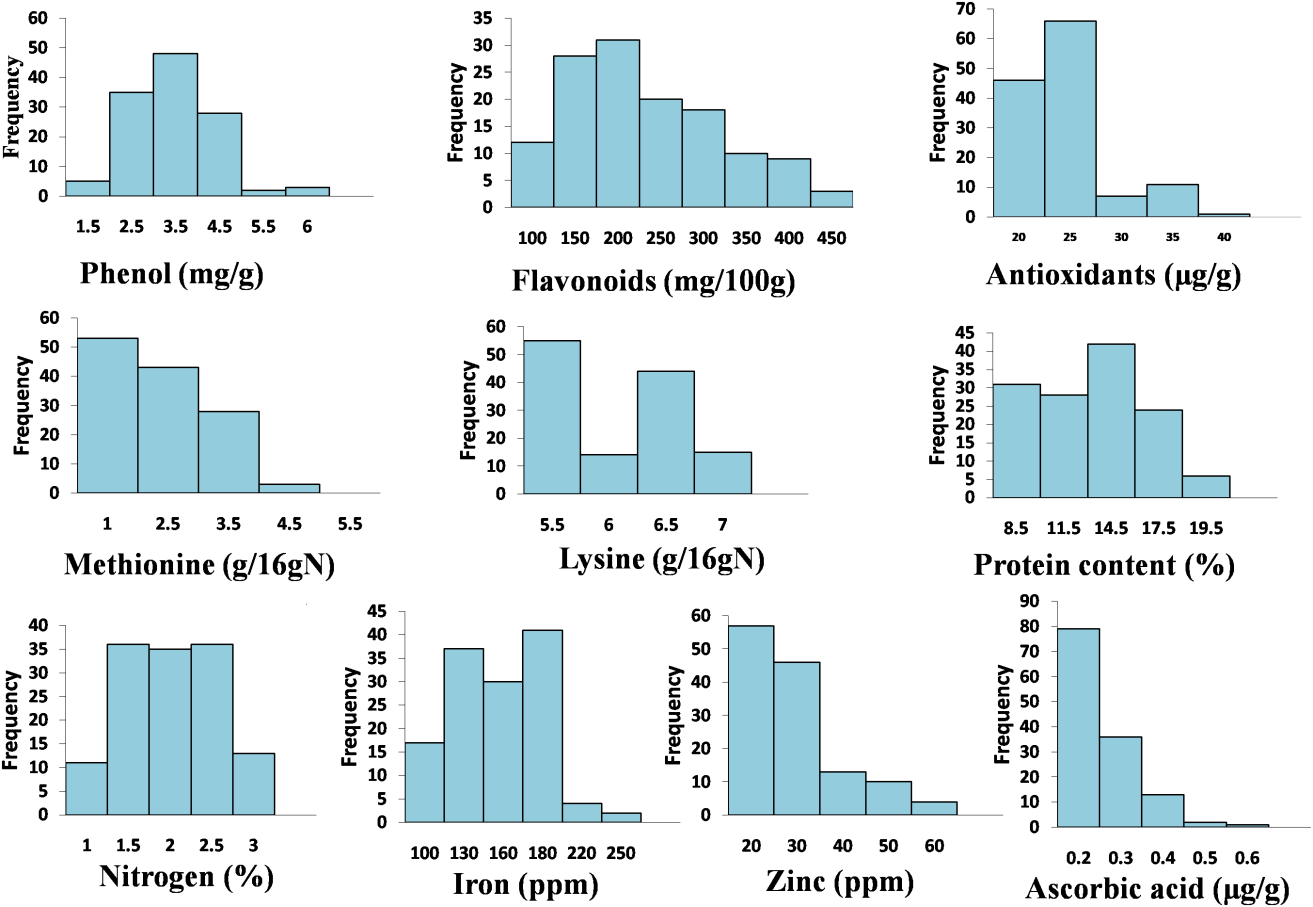
Histogram-based frequency distribution of 10 different nutri-nutraceutical components in buckwheat, i.e., phenol, flavonoid, antioxidants, methionine, lysine, protein content, nitrogen, iron, zinc, and ascorbic acid.

Nutritional and nutraceutical profiling of 132 buckwheat genotypes was carried out to study the variations and correlation among these traits with respect to each other. Buckwheat seed nutritional and nutraceutical profiling showed continuous variation among all these traits. These traits were estimated by different methods, and their mean values are shown in **Supplementary Table 2**. The range for each trait (lowest content to highest content) was recorded and showed huge variations. Phenol ranged from 0.54 to 5.85 mg/g; flavonoids, 65.08–475.5 mg/100 g; antioxidants, 17.23–37.53 µg/g; methionine, 1.09–4.71 g/16 g N; Lysine, 5.1–6.9 g/16 g N; protein, 5.8%–18.9%; nitrogen, 0.98%–2.90%; iron, 80.5–233.1 ppm; zinc, 12.78–57.70 ppm; and ascorbic acid, 0.02–0.60 µg/g. Among all the 132 buckwheat genotypes, phenol, flavonoids, antioxidants, iron, zinc, and nitrogen content was found high in *Fagopyrum tartaricum* (Bitter buckwheat) species, while methionine, lysine, protein, and ascorbic acid was found high in *F. esculentum* (common buckwheat) species. All the 10 nutritional traits for 132 genotypes with the highest and lowest performance (genotypes with the highest content and lowest content) are depicted in **Figure 1**. Very low coefficients of variation (CVs) were observed for all these nutritional and nutraceutical traits. Frequency distribution analysis was also done for all the 10 nutri-nutraceutical traits as represented in **Figures 2A**. These frequency distribution histograms explained the distribution of data by displaying the frequency of observations within different intervals. These frequency distribution histograms also show how often certain values occur within a dataset, giving a visual representation of the data spread and concentration.

### 3.2 Correlation analysis

**Figure 3:**
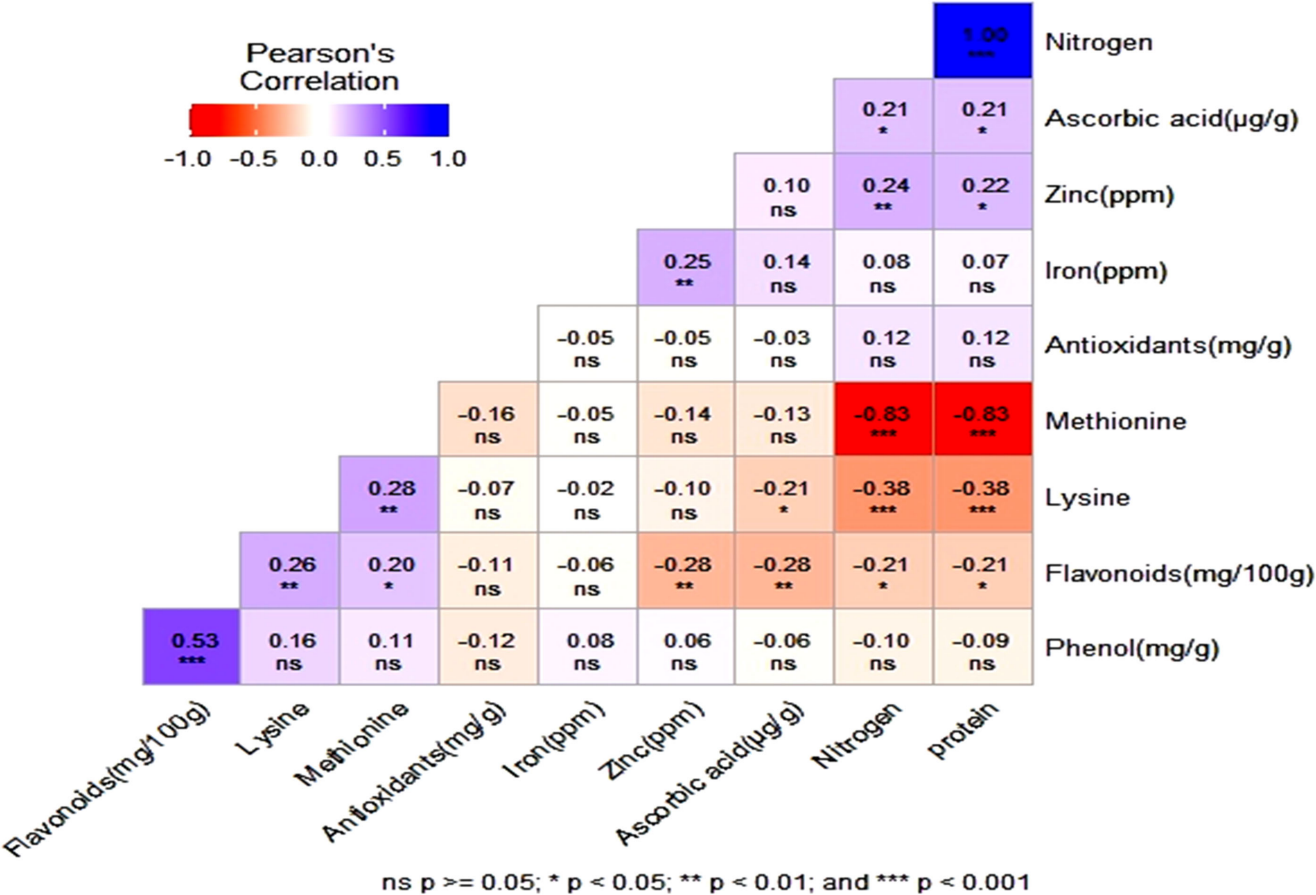
The heatmap showing the correlation between phenol, flavonoid, antioxidants, lysine, methionine, zinc, iron, nitrogen, protein, and ascorbic acid. ns, non significant.

The correlation heatmap displayed the correlation between multiple variables as a color-coded matrix. Each variable is represented by a row and a column and the cells show the correlation between them. The color of each cell represents the strength and direction of the correlation, with darker colors indicating stronger correlations. Pearson’s correlation analysis of 10 nutri-nutraceutical traits among 132 different genotypes showed positive correlation between the traits like nitrogen and protein, phenol and flavonoids, methionine and lysine, zinc and iron, flavonoids and lysine, zinc and protein, and ascorbic acid and nitrogen. However, methionine showed negative correlation with protein and nitrogen (**Figure 3**).

### 3.3 Characterization and chromosome-wise distribution of SNPs

A total of 4,142,684 variants were identified from 132 diverse genotypes, containing 3,728,028 SNPs and 414,656 InDels (214,798 insertions and 199,858 deletions). The highest number of SNPs (582,710) was observed on chromosome 1, whereas the lowest number of SNPs (362,087) was observed on chromosome 7. SNPs have also been classified into high, low, moderate, and modifier impact. SNP effects are categorized by impact as high (affecting splice-site, stop, and start codons), moderate (non-synonymous), low (synonymous coding/start/stop and start gained), and modifier (upstream, downstream, intergenic, and untranslated region) with the percentage of the high-impact SNPs being 0.413%; low, 1.566%; moderate, 2.105%; and modifier, 95.916%. According to the effects by functional class, missense percentage was 59.929%; nonsense, 3.3745%; and silent, 36.696%. Total number of transitions were 39,416,882 and total transversions were 23,590,338. The transition/transversion ratio (Ts/Tv) was 1.6709. Average SNP frequency per chromosome indicates the density of genetic variation within a given genomic region. This average SNP frequency is calculated by dividing the total number of SNPs by the length of the genomic region being analyzed, such as a chromosome (**Supplementary Figure 1**).

### 3.4 Genetic diversity and population structure

**Figure 4:**
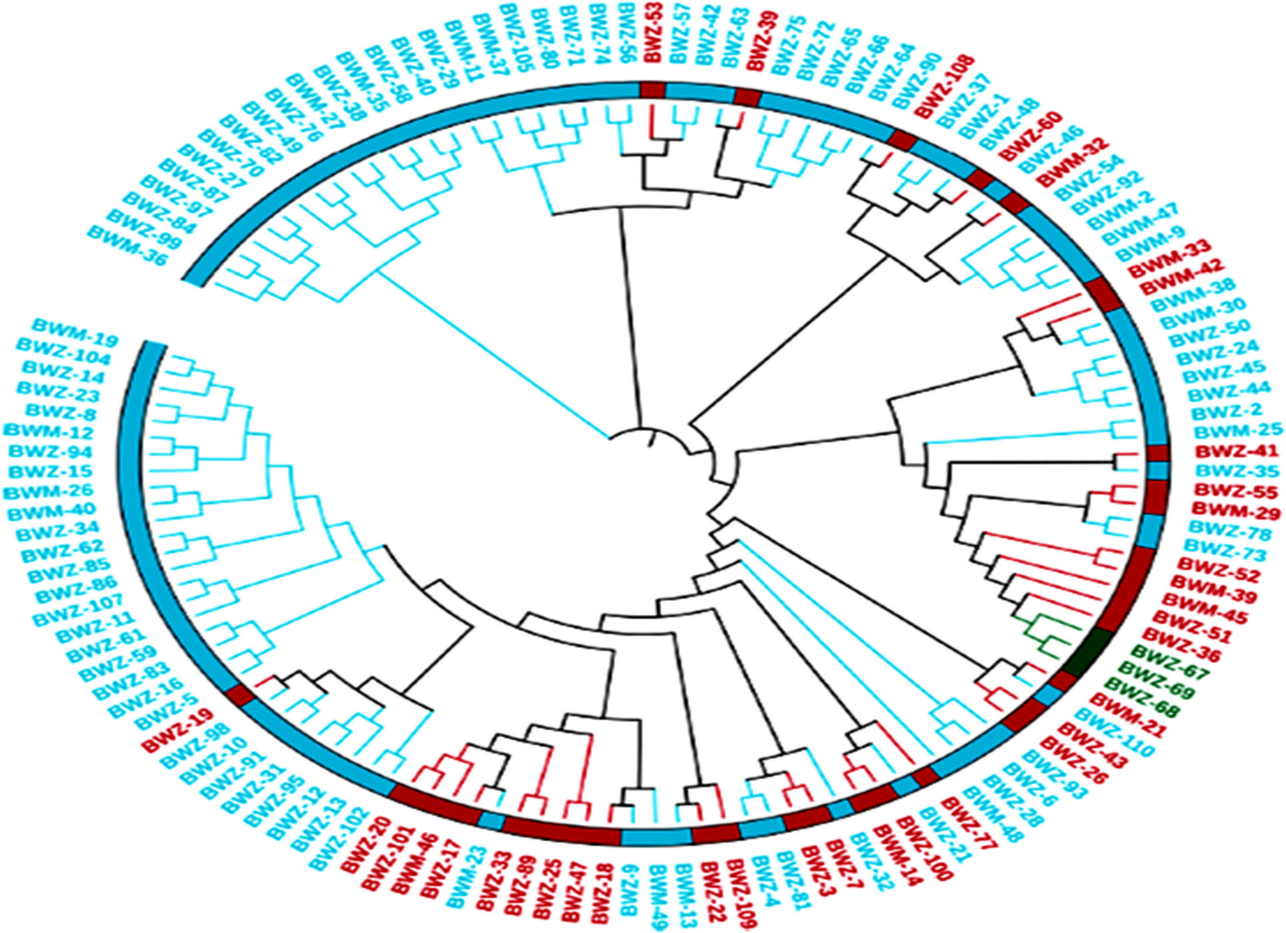
SNP marker-based population relationship among genotypes. The unweighted pair group method with arithmetic mean (UPGMA) dendrogram showing the genetic relationship among 132 diverse buckwheat genotypes.

The genetic distances between the 132 buckwheat accessions were determined from SNP-based genotypic data. A neighboring tree based on these genetic distances showed that the genotypes were divided into four main groups and were further divided into subgroups (**Figure 4**). PCA also showed diversity among the buckwheat genotypes. In addition, population structure analysis provides a robust analysis for understanding the genotypic origins of a particular crop. The population structure was scored for *K*-values ranging from 2 to 7 across the panel using high-quality SNPs. The peak of delta *K* was found to be the highest at *K* = 3 and thus grouped the 132 genotypes of buckwheat into three populations (**Supplementary Figure 2**).

### 3.5 Marker–trait association

**Figure 5:**
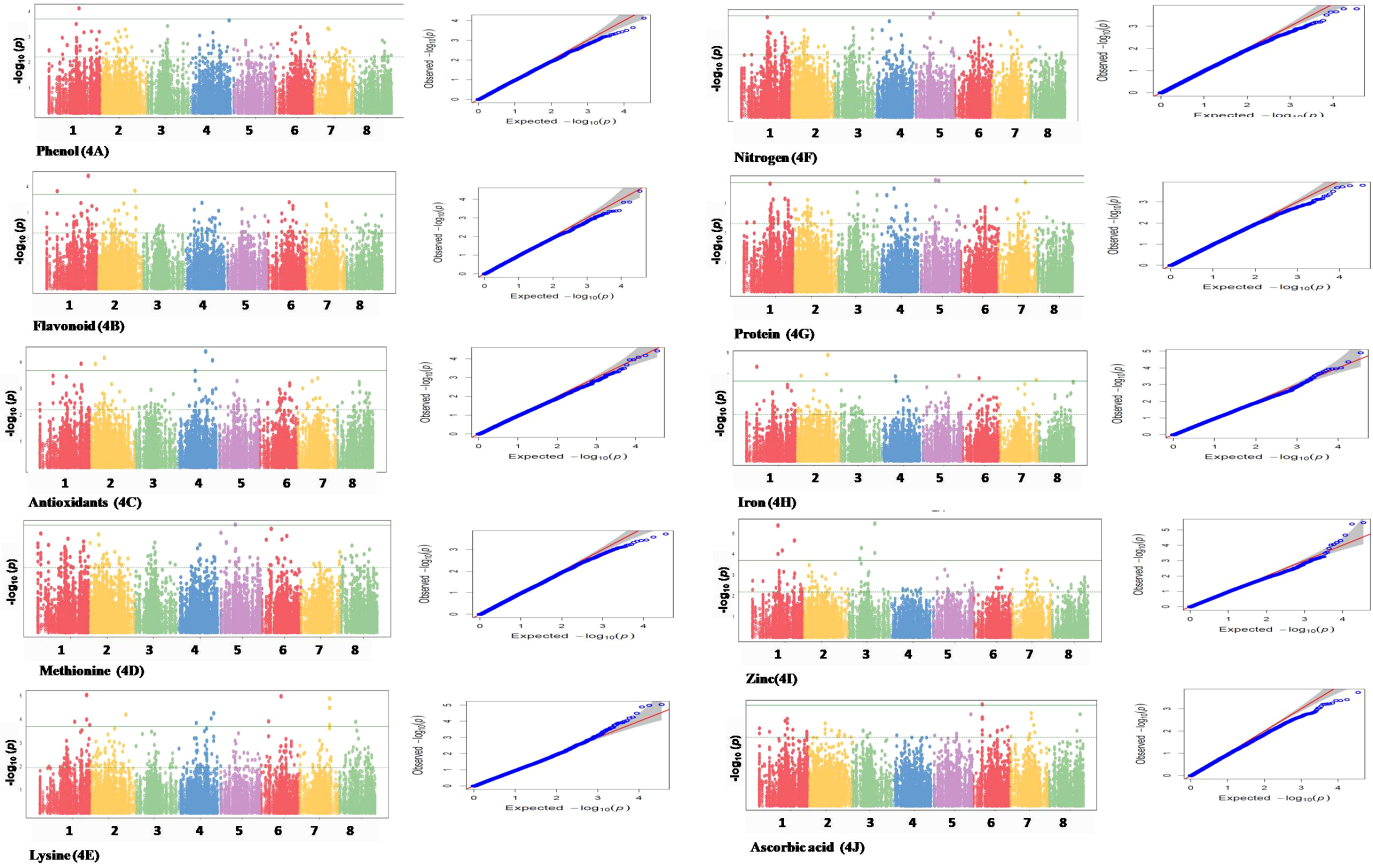
Manhattan and QQ plots of different marker associations with nutri-nutraceutical traits in buckwheat.

**Figure 6:**
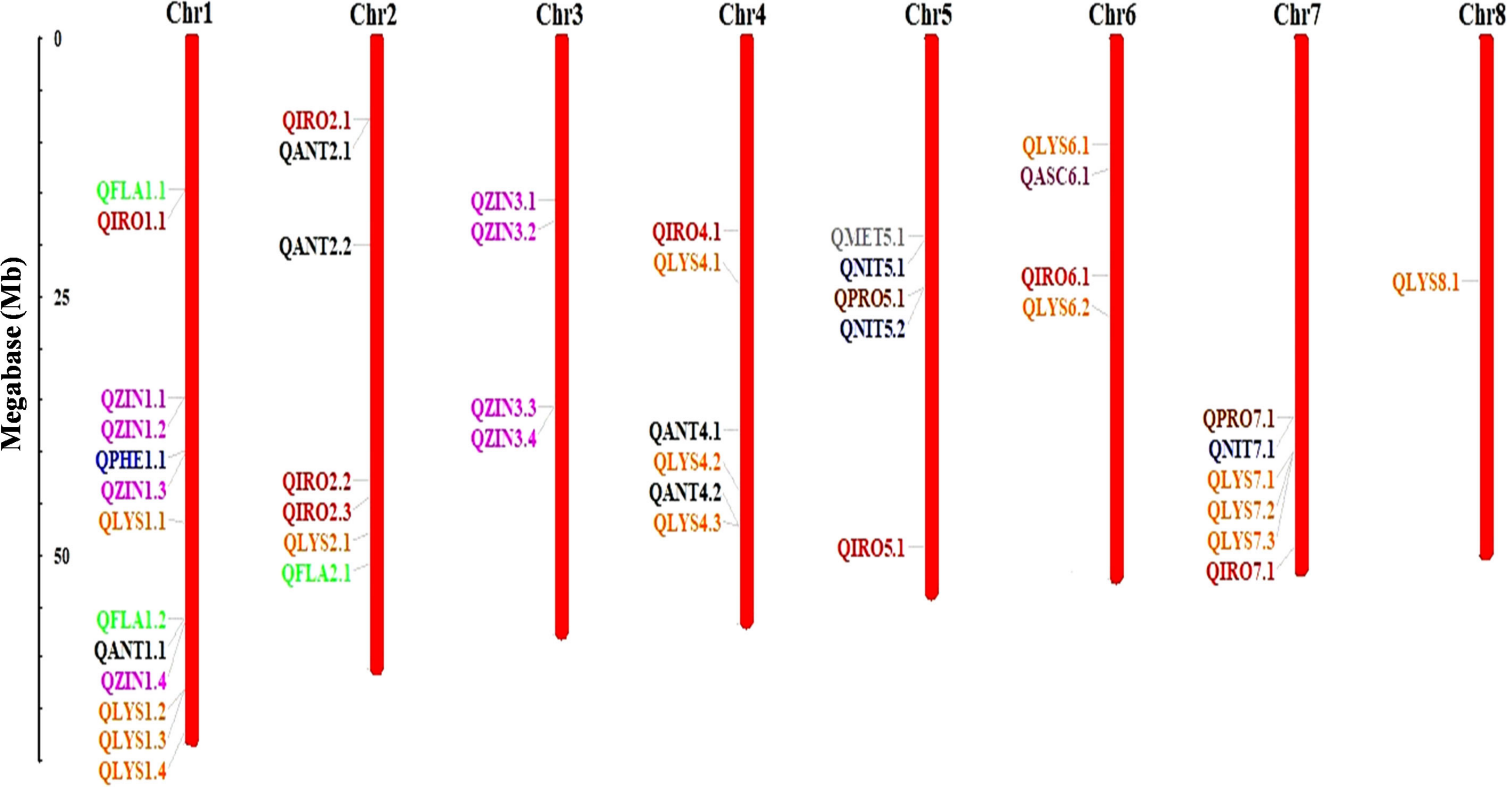
Chromosomal localization and distribution of quantitative trait loci (QTLs) in buckwheat. Position of each QTL is indicated by line, whereas scale bar represents the length of chromosomes in megabases (Mb).

GWAS was performed for 10 seed nutri-nutraceutical traits: phenol, flavonoid, antioxidants, methionine, lysine, nitrogen, protein content, iron, zinc, and ascorbic acid. With these traits, 46 SNPs were found having significant association. The details of these significant SNPs/marker–trait associations (MTAs) are summarized in **Table 1** and depicted in Manhattan and QQ plots (**Figure 5**). The QQ plots illustrate the observed association between markers and phenotype of interest (POI) compared to expected association after accounting for population structure. For phenol, a single SNP was found significantly associated on chromosome 1. This single SNP on chromosome 1 positioned at 40004476 (*p*-value = 0.00019) contributed 27.27% phenotypic variation. For flavonoid, three SNPs were found significantly associated on chromosomes 1 and 2. One SNP on chromosome 2 positioned at 50997418 (*p*-value = 0.000144) contributed 35.98% phenotypic variation. In the case of antioxidants, five SNPs were found significantly associated on chromosomes 1, 2, and 4. One SNP on chromosome 2 positioned at 19941896 (*p*-value = 6.73E−05) contributed 42.04% phenotypic variation. A total of 14 SNPs were found associated with the amino acid lysine that are positioned on chromosomes 1, 2, 4, 6, 7, and 8. Among these 14 SNPs, the highest number of SNPs, i.e., 4, associated with lysine was found on chromosome 1. One SNP on chromosome 6 positioned at 26920730 (*p*-value = 1.05E-05) contributed 49.62% of phenotypic variation. For methionine, only one SNP was found significantly associated on chromosome 5. This single SNP on chromosome 5 positioned at 19139581 (*p*-value = 0.00019) contributed 32.95% phenotypic variation. For nitrogen, three SNPs that are positioned on chromosomes 5 and 7 were found significantly associated. One SNP on chromosome 5 positioned at 24057542 (*p*-value = 0.000172) contributed 23.86% phenotypic variation. For protein, two SNPs were found significantly associated on chromosomes 5 and 7. One SNP on chromosome 5 positioned at 24057542 (*p*-value = 0.000166) contributed 23.86% phenotypic variation. For iron, eight SNPs that are positioned on chromosomes 1, 2, 4, 5, 6, and 7 were found significantly associated. For zinc, eight SNPs that are positioned on chromosomes 1 and 3 were found significantly associated, with an equal number of SNPs each on chromosomes 1 and 3. One SNP on chromosome 3 positioned at 35689620 (*p*-value = 3.40E−06) contributed 20.79% phenotypic variation. For ascorbic acid, a single SNP was found significantly associated on chromosome 6. The genome location of 46 significant SNPs for different nutritional and nutraceutical traits of buckwheat was generated by map inspect software (**Figure 6**).

**Table 1:** The list of significant SNPs/QTLs along with candidate genes for 10 nutritional and nutraceutical traits of buckwheat.

Traits	QTLs	Chromosome	SNP Position	*P*-value	Minor allele frequency (MAF)	Start	End	Candidate Genes ID
Phenol	QPHN1.1	1	40004476	7.59E−05	0.272	39954476	40054476	FtPinG0007197200.01, FtPinG0007196500.01, FtPinG0007196400.01, FtPinG0007196900.01, FtPinG0007196800.01
Flavonoid	QFLA1.1	1	14639543	0.000149034	0.143	14589543	14689543	FtPinG0005599700.01, FtPinG0005599400.01, FtPinG0005601600.01, FtPinG0005602100.01
	QFLA1.2	1	56206331	3.75E−05	0.189	56156331	56256331	FtPinG0009546300.01, FtPinG0009547000.01, FtPinG0009546100.01, FtPinG0009546600.01
	QFLA2.1	2	50997418	0.000144117	0.359	50947418	51047418	FtPinG0006999000.01, FtPinG0006999200.01, FtPinG0006999300.01
Antioxidant	QANT1.1	1	56207465	0.000113025	0.151	56157465	56257465	FtPinG0009546300.01, FtPinG0009547000.01, FtPinG0009546100.01, FtPinG0009546600.01
	QANT2.1	2	7855102	0.000116183	0.087	7805102	7905102	FtPinG0009542600.01, FtPinG0009541700.01, FtPinG0001547800.01, FtPinG0009542100.01
	QANT2.2	2	19941896	6.73E−05	0.420	19891896	19991896	FtPinG0000053600.01
	QANT4.1	4	37948541	3.87E−05	0.132	37898541	37998541	FtPinG0006787400.01, FtPinG0006787600.01, FtPinG0004996400.01, FtPinG0006787100.01
	QANT4.2	4	47186890	8.41E−05	0.166	47136890	47236890	FtPinG0005258300.01, FtPinG0005259000.01, FtPinG0007507100.01, FtPinG0005258100.01, FtPinG0005258700.01
Methionine	QMET5.1	5	19139581	0.000189768	0.329	19089581	19189581	FtPinG00034919.01
Lysine	QLYS1.1	1	47054068	0.000126124	0.201	47004068	47104068	FtPinG0006245400.01, FtPinG0006244900.01
	QLYS1.2	1	63056676	9.31E−06	0.140	63006676	63106676	FtPinG0007146800.01, FtPinG0007147500.01, FtPinG0007147100.01, FtPinG0007147800.01, FtPinG0007146900.01
	QLYS1.3	1	63056706	0.000102386	0.162	63006706	63106706	FtPinG0007146800.01, FtPinG0007147500.01, FtPinG0007147100.01, FtPinG0007147800.01, FtPinG0007146900.01
	QLYS1.4	1	67182755	0.000175398	0.106	67132755	67232755	FtPinG0007906400.01, FtPinG0007907500.01, FtPinG0007911300.01, FtPinG0007909900.01, FtPinG0007905200.01, FtPinG0007908700.01
	QLYS2.1	2	48025684	6.32E−05	0.268	47975684	48075684	FtPinG0001529700.01, FtPinG0001529600.01, FtPinG0001529300.01, FtPinG0001530000.01
	QLYS4.1	4	23778598	0.000143468	0.284	23728598	23828598	FtPinG0009843200.01
	QLYS4.2	4	43826569	9.23E−05	0.238	43776569	43876569	FinG0001537500.01, FtPinG0001538600.01, FtPinG0001538200.01, FtPinG0001538800.01, FtPinG0001537000.01, FtPinG0001538300.01
	QLYS4.3	4	47186890	5.54E−05	0.166	47136890	47236890	FtPinG0005258300.01, FtPinG0005259000.01, FtPinG0007507100.01, FtPinG0005258100.01, FtPinG0005258700.01
	QLYS6.1	6	10161864	0.000122356	0.208	10111864	10211864	FtPinG0007535500.01, FtPinG0007534900.01, FtPinG0007534300.01, FtPinG0007534500.01, FtPinG0007535200.01, FtPinG0007534800.01, FtPinG0007535700.01, FtPinG0007535300.01, FtPinG0007535900.01, FtPinG0007536100.01
	QLYS6.2	6	26920730	1.05E−05	0.496	26870730	26970730	FtPinG0008711500.01, FtPinG0003067000.01
	QLYS7.1	7	40000527	3.23E−05	0.143	39950527	40050527	FtPinG0005842300.01, FtPinG0005842700.01
	QLYS7.2	7	40000561	0.000169696	0.132	39950561	40050561	FtPinG0005842300.01, FtPinG0005842700.01
	QLYS7.3	7	40000586	1.30E−05	0.121	39950586	40050586	FtPinG0005842300.01, FtPinG0005842700.01
	QLYS8.1	8	23398412	0.000128189	0.196	23348412	23448412	FtPinG0006164000.01, FtPinG0006160900.01, FtPinG0006163100.01, FtPinG0006164200.01, FtPinG0006161800.01, FtPinG0006163600.01
Protein	QPRO5.1	5	24057542	0.000165662	0.238	24007542	24107542	FtPinG0005685000.01, FtPinG0005685100.01
	QPRO7.1	7	36559176	0.000165418	0.170	36509176	36609176	FtPinG0006636800.01, FtPinG0008784600.01, FtPinG0008785000.01, FtPinG0006637600.01, FtPinG0006636600.01, FtPinG0006637400.01
Nitrogen	QNIT5.1	5	19640595	0.00016449	0.208	19590595	19690595	FtPinG0004866200.01, FtPinG0004865100.01, FtPinG0004866000.01, FtPinG0004865400.01, FtPinG0004865700.01
	QNIT5.2	5	24057542	0.000172165	0.238	24007542	24107542	FtPinG0005685000.01, FtPinG0005685100.01
	QNIT7.1	7	36559176	0.000195294	0.170	36509176	36609176	FtPinG0006636800.01, FtPinG0008784600.01, FtPinG0008785000.01, FtPinG0006637600.01, FtPinG0006636600.01, FtPinG0006637400.01
Iron	QIRO1.1	1	14675782	4.39E−05	0.215	14625782	14725782	FtPinG0005599700.01, FtPinG0004691300.01, FtPinG0005599400.01
	QIRO2.1	2	7853900	0.000114287	0.117	7803900	7903900	FtPinG0009542600.01, FtPinG0009541700.01, FtPinG0001547800.01, FtPinG0009542100.01
	QIRO2.2	2	42749461	9.89E−05	0.170	42699461	42799461	FtPinG0004496500.01, FtPinG0004496400.01, FtPinG0004496700.01
	QIRO2.3	2	44458379	1.26E−05	0.299	44408379	44508379	FtPinG0006324700.01, FtPinG0006324300.01, FtPinG0006323500.01, FtPinG0006323700.01, FtPinG0006324000.01, FtPinG0006324200.01
	QIRO4.1	4	18606963	0.000120993	0.151	18556963	18656963	FtPinG0003491500.01, FtPinG0003491700.01, FtPinG0003492100.01, FtPinG0003491900.01
	QIRO5.1	5	49199435	0.000115213	0.212	49149435	49249435	FtPinG0006495600.01, FtPinG0006496800.01, FtPinG0006362500.01, FtPinG0006496100.01, FtPinG0006495900.01, FtPinG0006496300.01
	QIRO6.1	6	22925002	0.000146404	0.208	22875002	22975002	FtPinG0006104000.01, FtPinG0006103300.01, FtPinG0006103100.01, FtPinG0006104100.01
	QIRO7.1	7	49444717	0.000174248	0.201	49394717	49494717	FtPinG0003509300.01, FtPinG0003509800.01, FtPinG0003508600.01, FtPinG0003507700.01, FtPinG0003507500.01, FtPinG0003509500.01
Zinc	QZIN1.1	1	34715704	9.78E−05	0.306	34665704	34765704	FtPinG0004724200.01, FtPinG0004725500.01, FtPinG0004725400.01, FtPinG0006706600.01, FtPinG0004726000.01, FtPinG0004724400.01
	QZIN1.2	1	34715713	4.20E−06	0.295	34665713	34765713	FtPinG0004724200.01, FtPinG0004725500.01, FtPinG0004725400.01, FtPinG0006706600.01, FtPinG0004726000.01, FtPinG0004724400.01
	QZIN1.3	1	40493945	6.68E−05	0.151	40443945	40543945	FtPinG0009458700.01, FtPinG0006043600.01, FtPinG0009458400.01, FtPinG0009459500.01, FtPinG0009459200.01, FtPinG0009458500.01
	QZIN1.4	1	56716710	2.22E−05	0.201	56666710	56766710	–
	QZIN3.1	3	15675077	0.000162978	0.147	15625077	15725077	FtPinG0008630900.01, FtPinG0008630500.01, FtPinG0008631200.01, FtPinG0008630300.01, FtPinG0008630800.01, FtPinG0008631700.01, FtPinG0008631900.01, FtPinG0008631600.01
	QZIN3.2	3	17689541	5.03E−05	0.181	17639541	17739541	FtPinG0006132400.01
	QZIN3.3	3	35689615	8.80E−05	0.201	35639615	35739615	FtPinG0005355100.01, FtPinG0005354800.01, FtPinG0005355500.01, FtPinG0005355700.01, FtPinG0005355000.01, FtPinG0005355300.01
	QZIN3.4	3	35689620	3.40E−06	0.204	35639620	35739620	FtPinG0005355100.01, FtPinG0005354800.01, FtPinG0005355500.01, FtPinG0005355700.01, FtPinG0005355000.01, FtPinG0005355300.01
Ascorbic acid	QASC6.1	6	12715547	0.000187	0.122	12665547	12765547	FtPinG0007082400.01, FtPinG0007082600.01, FtPinG0007081300.01, FtPinG0007083200.01, FtPinG0007081800.01, FtPinG0007083500.01, FtPinG0007081000.01, FtPinG0007081200.01

### 3.6 LD plot

**Figure 7:**
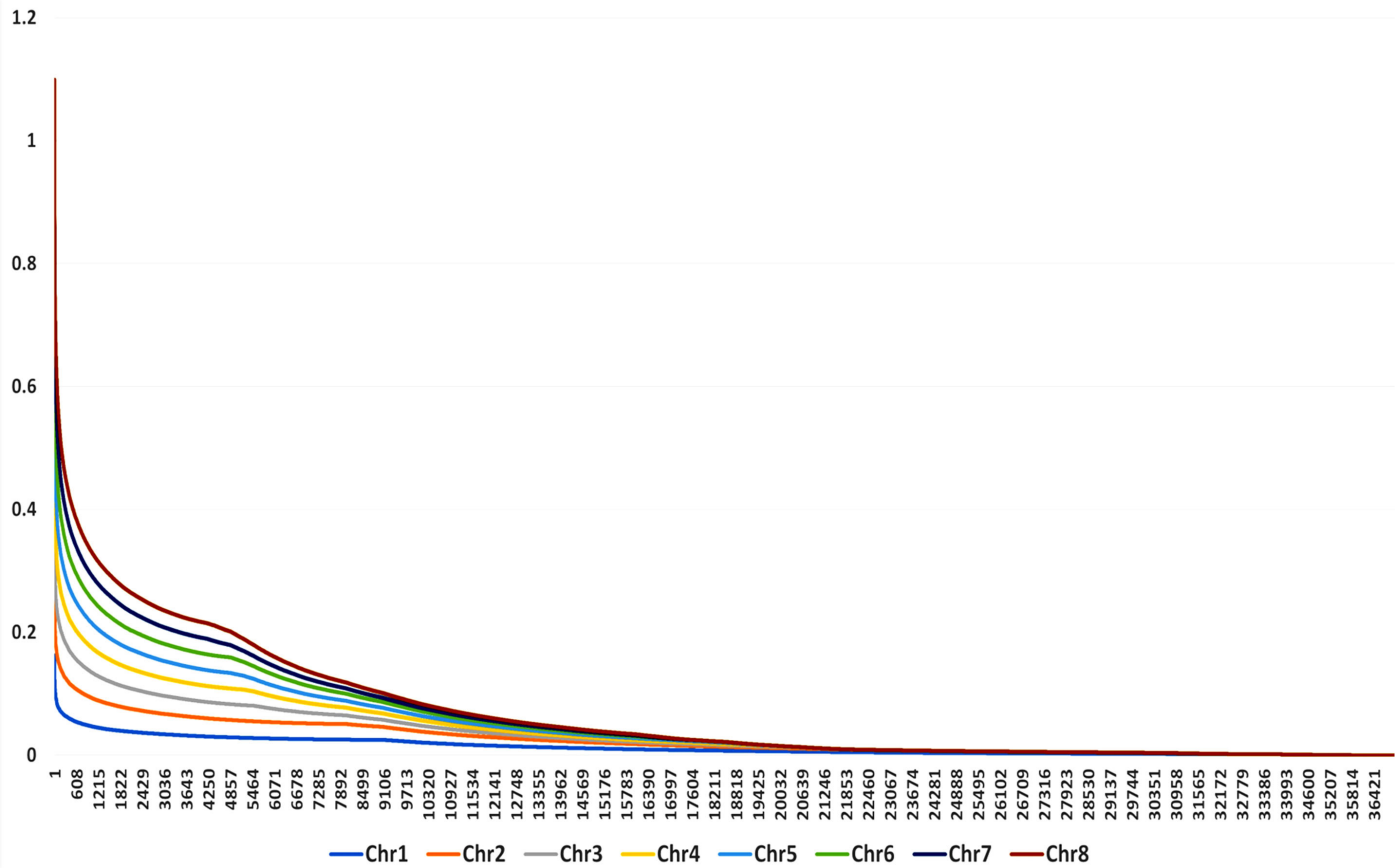
Combined LD plot for 132 genotypes of buckwheat. The *X*-axis represents the physical position of genetic variants along the chromosome, and the *Y*-axis represents the measure of LD between pairs of genetic variants.

LD was calculated from 4,142,684 pairs using a 100-marker sliding window operation, out of which 8% had zero LD and 23% was found in the significant range (*p*-value < 0.05). As the physical distance increases, the *r* ^2^ distribution showed a rapid LD decay for all genotypes. LD values for each chromosome is represented by color gradient with different colors indicating different levels of LD. Chromosomes with red shades represent high LD, while chromosomes with blue or green shade represent low LD. In the combined LD plot, chromosome 8 represents high LD and chromosome 1 represents low LD (**Figure 7**).

### 3.7 Candidate gene identification

To further reveal the molecular function of the SNPs significantly associated with the 10 different nutritional and nutraceutical traits, the genes within the 100 kb upstream and downstream regions of the significant QTLs were also extracted. A 100-kb region is typically large enough to encompass multiple genes and their surrounding regulatory regions. This allowed us to explore the potential effects of genetic variants on nearby genes and regulatory elements, providing a broader understanding of the genetic architecture underlying the trait of interest. Moreover, LD between genetic variants tends to decay with increasing physical distance along the genome; thus, by focusing on a 100-kb region, we were able to capture genetic variants that were likely to be in LD with each other and were associated within the trait. A total of 138 candidate genes were identified. These candidate genes were found involved in various biological and molecular functions such as lipid metabolic process, carbohydrate metabolic process, and zinc ion binding. The 46 different SNPs along with the candidate genes associated with them are enlisted in **Table 1**. Genomic variants and gene annotations of buckwheat are provided in **Supplementary Tables 3** and **4**.

### 3.8 Gene Ontology analysis

GO analysis was performed to gain insight into the biological process being affected by candidate genes identified close to the significant SNPs associated with traits of interest. GO analysis divided annotation terms into biological process (BP), cellular components (CC), and molecular function (MF). The candidate genes identified associated with significant SNPs with various traits recorded are involved in biological processes like glycolytic process, carbohydrate metabolic process, RNA processing, protein methylation, and translation (**Supplementary Figure 3**). However, in the case of molecular functions, the gene coding for DNA, RNA, and protein binding activity was significantly enriched in the dataset (**Supplementary Figure 4**). According to GO analysis, the key biological processes involved are amino acid metabolic process, protein import into mitochondrial matrix, lipid transport, tryptophan metabolic process, carbohydrate metabolic process, DNA integration, RNA processing, protein phosphorylation, transmembrane transport, regulation of DNA-templated transcription, and biosynthetic process. The key molecular functions of the identified candidate genes were ATP binding, hydrolase activity, methyltransferase activity, acyltransferase activity, catalytic activity, nucleic acid binding, magnesium ion binding, phosphoprotein phosphate activity, DNA-binding transcription factor activity, and protein kinase activity. However, further *in vitro* validation of candidate genes is needed for establishing the proof of concept.

## 4 Discussion

The present study was a first attempt to utilize diverse buckwheat genotypes, facilitating the identification of novel SNP loci associated with 10 nutri-nutraceutical traits (phenol, flavonoids, antioxidants, methionine, lysine, protein content, nitrogen, iron, zinc, and ascorbic acid). These findings offer significant implications for the development of molecular markers to accelerate genomics-led breeding efforts and providing new insights into the genetic architecture regulating these traits of interest in buckwheat. Furthermore, this analysis could facilitate the identification of genetically diverse parental lines to improve nutritional and agronomic traits, contributing to strategies aimed at addressing malnutrition.

The evaluation of nutritional and nutraceutical traits revealed considerable variation among the 132 genotypes. Pearson’s correlation analysis revealed significant positive correlations between protein, nitrogen, flavonoids, phenol, zinc, and iron, indicating that the simultaneous selection of these traits could enhance the quality of this crop and can help breeders in utilizing correlated responses in the selection process. The nutritionally relevant bioactive compounds such as phenols, flavonoids, and antioxidants play a significant protective role in promoting human health by mainly preventing major lifestyle-related ailments.

Moreover, huge variations were observed in phenol content (0.54 ± 0.06 mg/g to 5.85 ± 0.005 mg/g), flavonoids (65.08 ± 0.97 mg/100 g to 475.5 ± 0.99 mg/100 g), and antioxidants (17.23 ± 0.045 µg/g to 37.53 ± 0.081 µg/g). These results align with previous studies (Inglett et al., 2010; Zielińska et al., 2012a; Hua et al., 2014) and highlight the genetic potential for enhancing these traits through breeding. In the case of amino acids, the levels of methionine (1.09 ± 0.05 g/16 g N to 4.71 ± 0.14 g/16 g N) and lysine (5.19 ± 0.06 g/16 g N to 6.91 ± 0.38 g/16 g N) vary moderately among the genotypes. However, the results are slightly higher but within the ranges reported in previous studies (Culetu et al., 2021a). These findings align with the known variation in protein content of *F. esculentum* and *F. tataricum* (Huda et al., 2021). Micronutrient analysis also revealed considerable variation, with some potential genotypes (BWM-39 and BWM-19) having high Fe and Zn possibly directly involved in further breeding programs.

GWASs have been widely used as a valuable tool in dissecting and understanding complex quantitative traits by identifying significant SNPs. However, it is often challenged by false positives due to unaccounted genetic structure and kinship, as well as false negatives from overly stringent statistical corrections (Zhang et al., 2017a). To address these issues, this study employed a CMLM via the GAPIT software and applied a false discovery rate (FDR) approach. This method balances the trade-off between false positives and false negatives more effectively than the conservative Bonferroni correction (Skrabanja et al., 2001). Previous research (Zhang et al., 2017b; Pandey et al., 2020) also emphasized the necessity of accounting for population structure and kinship in GWAS to reduce erroneous correlations, which was successfully done in this study.

The present study identified 46 SNPs significantly associated with the 10 traits, with varying degrees of phenotypic variation explained by each SNP. Given the limited availability of MTA data for buckwheat, these findings represent a significant contribution to the understanding of its genetic basis. A single SNP found on chromosome 1 was strongly related to phenol content, accounting for 27.27% of the phenotypic variation. This conclusion is consistent with a previous study on the genetic control of phenolic chemicals, which provides antioxidant capabilities to buckwheat, hence increasing its health advantages (Zielińska et al., 2012b). Similarly, SNPs on chromosomes 1 and 2 were linked to flavonoid content, with one SNP accounting for 35.98% of the variance. Flavonoids play important roles in plant defense and human health; therefore, these findings are useful for breeding programs targeted at improving these compounds. Furthermore, 14 SNPs associated with lysine content across many chromosomes, including one on chromosome 6 that accounts for 49.62% of the variation, highlight its potential for altering amino acid composition. Similarly, an SNP on chromosome 5 linked to methionine concentration explained 32.95% of variation, indicating potential for biofortification. These results are comparable to those reported by Culetu et al. (2021b), who found significant genetic variation in amino acid content among buckwheat genotypes. A shared SNP on chromosome 5 accounted for 23.86% of variation in nitrogen and protein concentration, indicating that these variables are genetically controlled together. This result indicates that the genetic basis of protein and nitrogen content is linked, as evidenced by their positive correlation, and offers opportunities for simultaneous improvement through marker-assisted selection.

LD analysis found considerable levels of LD throughout the genome, with chromosome 8 having the highest level and chromosome 1 having the lowest. The observed LD decay pattern in this study aligns with findings in related cereal and pseudocereals. In rice (*Oryza sativa*), LD decay has been reported to occur within 6–8 cM across cultivated varieties, indicating a moderate rate of decay in this self-pollinating species (Ouyang et al., 2011). Similarly, in quinoa (*Chenopodium quinoa*), a pseudocereal, LD decay between SNPs was found to be 32.4 kb, suggesting a relatively slower decay compared to maize (Ruiz et al., 2020).

Moreover, the present study also identified certain candidate genes that lie within the 0.1-Mb flanking regions of significant SNPs/QTLs associated with various traits. Our analysis highlighted key metabolic and biosynthetic pathways, including butanoate metabolism, glycerophospholipids, arachidonic acid, and glutathione, as well as the biosynthesis of flavonoids, sesquiterpenoids, and triterpenoids. These pathways contribute to stress resilience and play roles in amino acid metabolism, energy processes, and antioxidant defense (**Supplementary Figures 1** and **2**). The identified candidate genes provide molecular resources for future studies, with potential applications in molecular breeding, marker-assisted selection, and gene editing.

## 5 Conclusion

The increasing appreciation for the value of healthy and balanced diets for promoting better human health has led to increased demand for foods with nutritional and nutraceutical values. Buckwheat is an underutilized crop but holds tremendous promise as an alternative diet to staple cereals on account of its nearly perfect composition. In order to introduce this valuable crop in existing farming systems, genetic resources and OMICS tools can enhance the understanding of nutraceutical value and identify genotypes that can competitively be aligned with major cereal crops. The SNP markers and candidate genes revealed in this study can help establish a dependable molecular marker-based selection method to speed up conventional breeding efforts. Early identification of desired genotypes will simplify breeding and conserve resources. Buckwheat, known for its nutritional value and drought resistance, has the potential for better nutritional features via GWAS. This research can serve to influence breeding efforts, improve the nutritional value of buckwheat, combat malnutrition, and promote sustainable food systems, all of which enhance world health and food security.

## Data Availability

The datasets presented in this study can be found in online repositories. The names of the repository/repositories and accession number(s) can be found below: https://www.ncbi.nlm.nih.gov/, PRJNA996322.
