# Supplementary material for: Genome-wide identification and association analysis of informative SNPs of various nutri-nutraceutical traits in Buckwheat (Fagopyrum spp.)
Source: Front Plant Sci. 2025 Apr 24;16:1559621. doi: 10.3389/fpls.2025.1559621 (PMC12059574; doi:10.3389/fpls.2025.1559621)
Supplement: Supplementary file 1 [file SupplementaryFile1.zip › Supplementary Material/Supplementary Figures 1-4.pptx]

## Slide 1
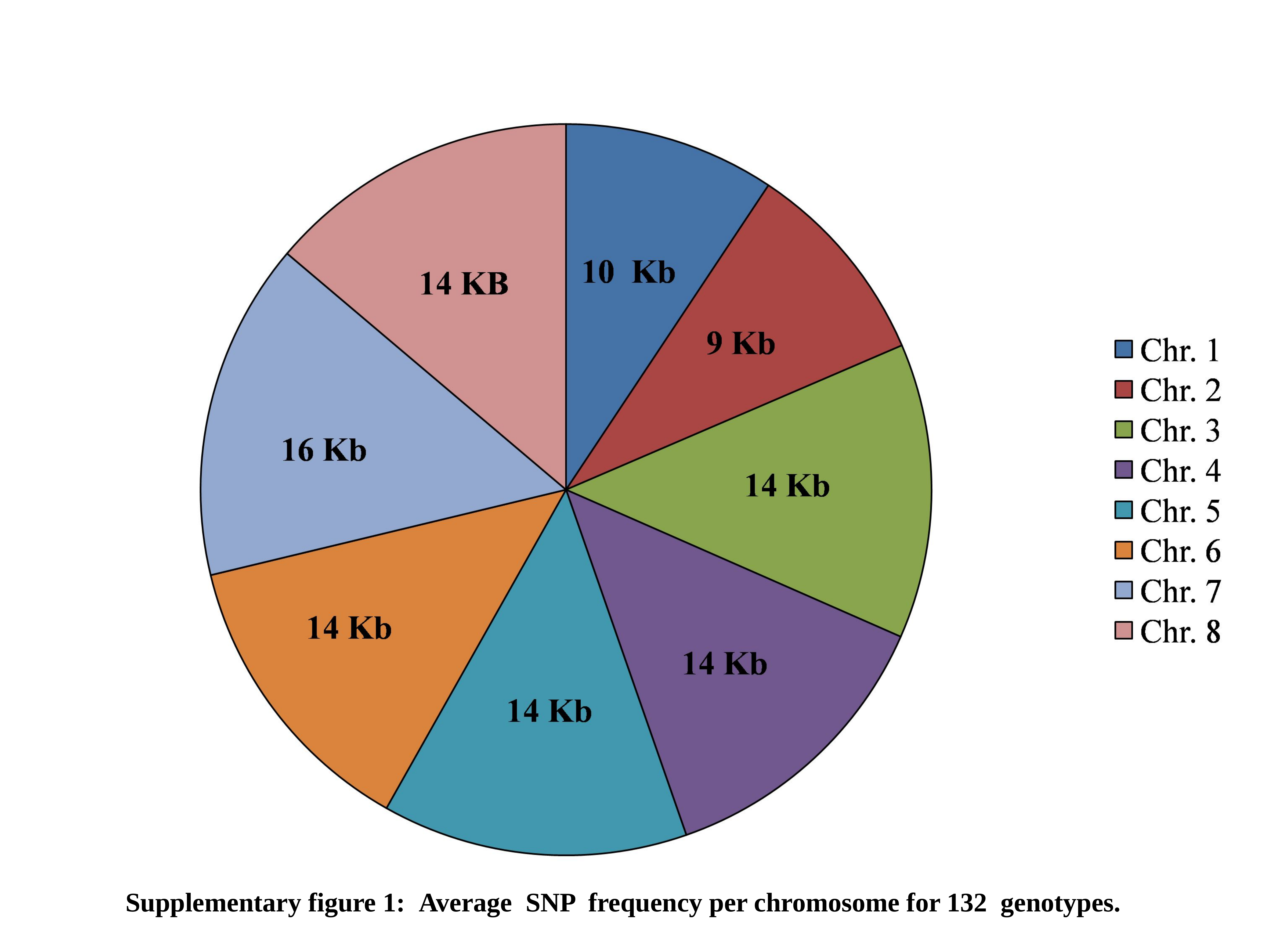

Supplementary figure 1: Average SNP frequency per chromosome for 132 genotypes.

## Slide 2
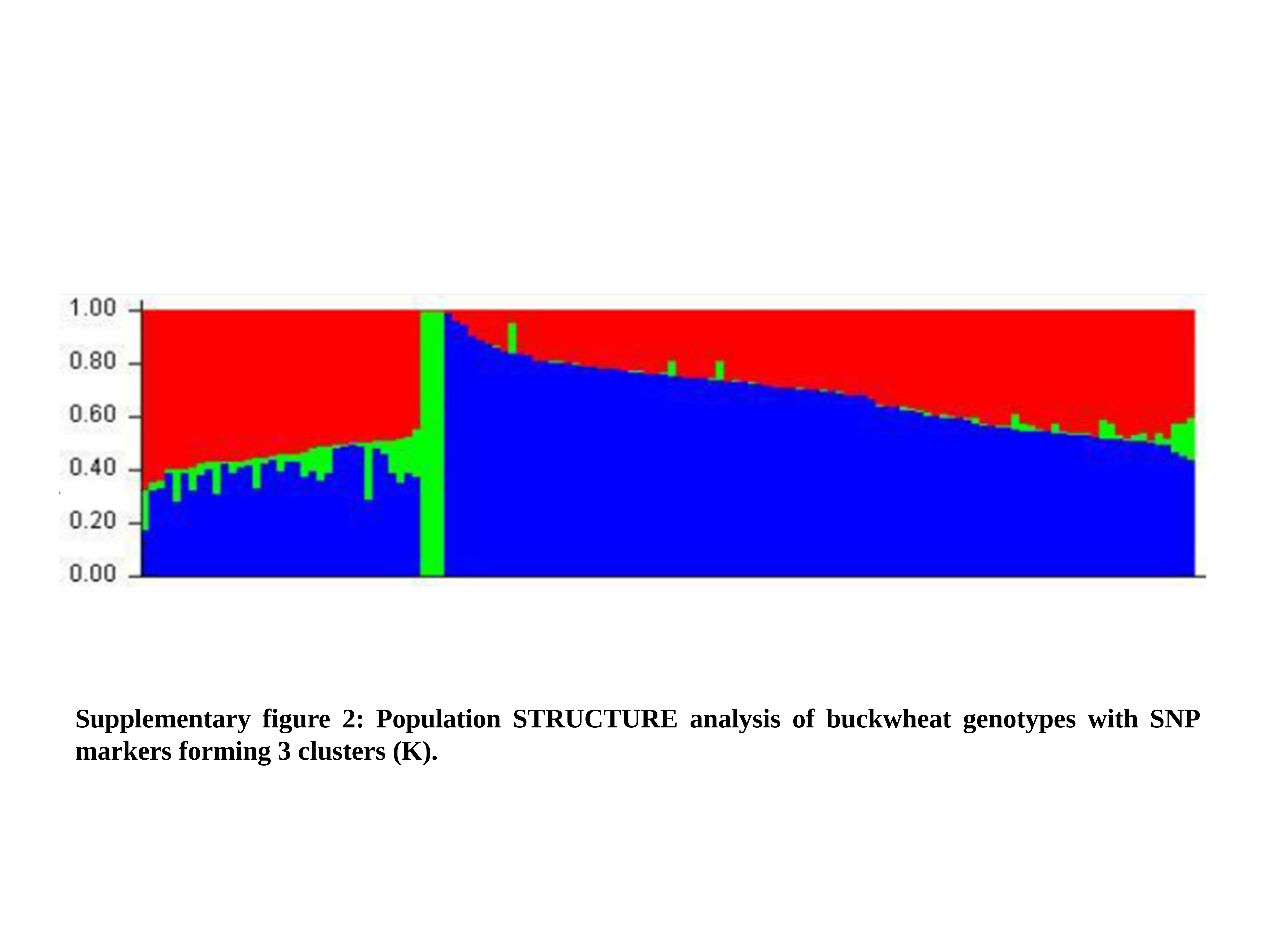

Supplementary figure 2: Population STRUCTURE analysis of buckwheat genotypes with SNP markers forming 3 clusters (K).

## Slide 3
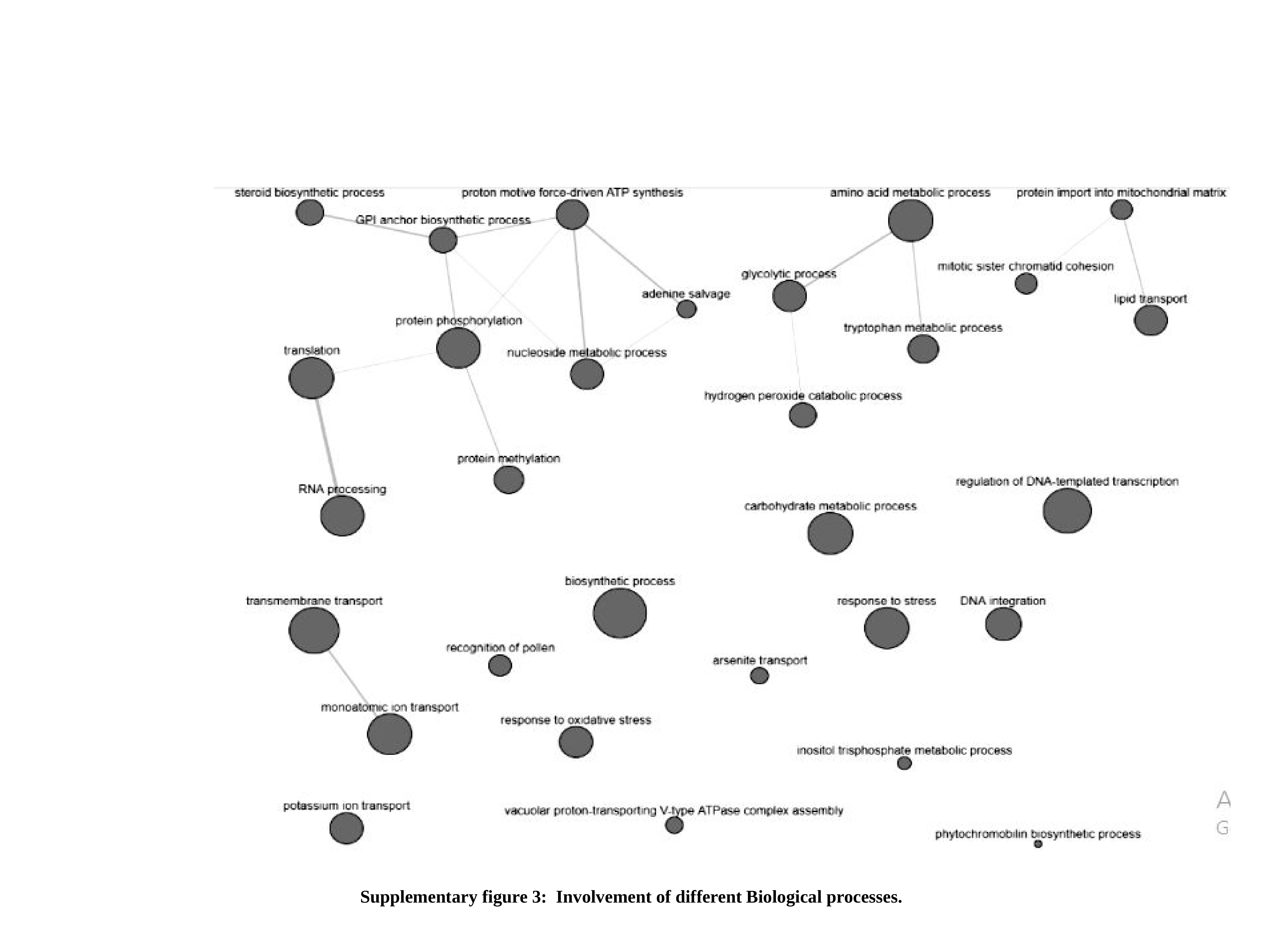

# Supplementary figure 3: Involvement of different Biological processes.

## Slide 4
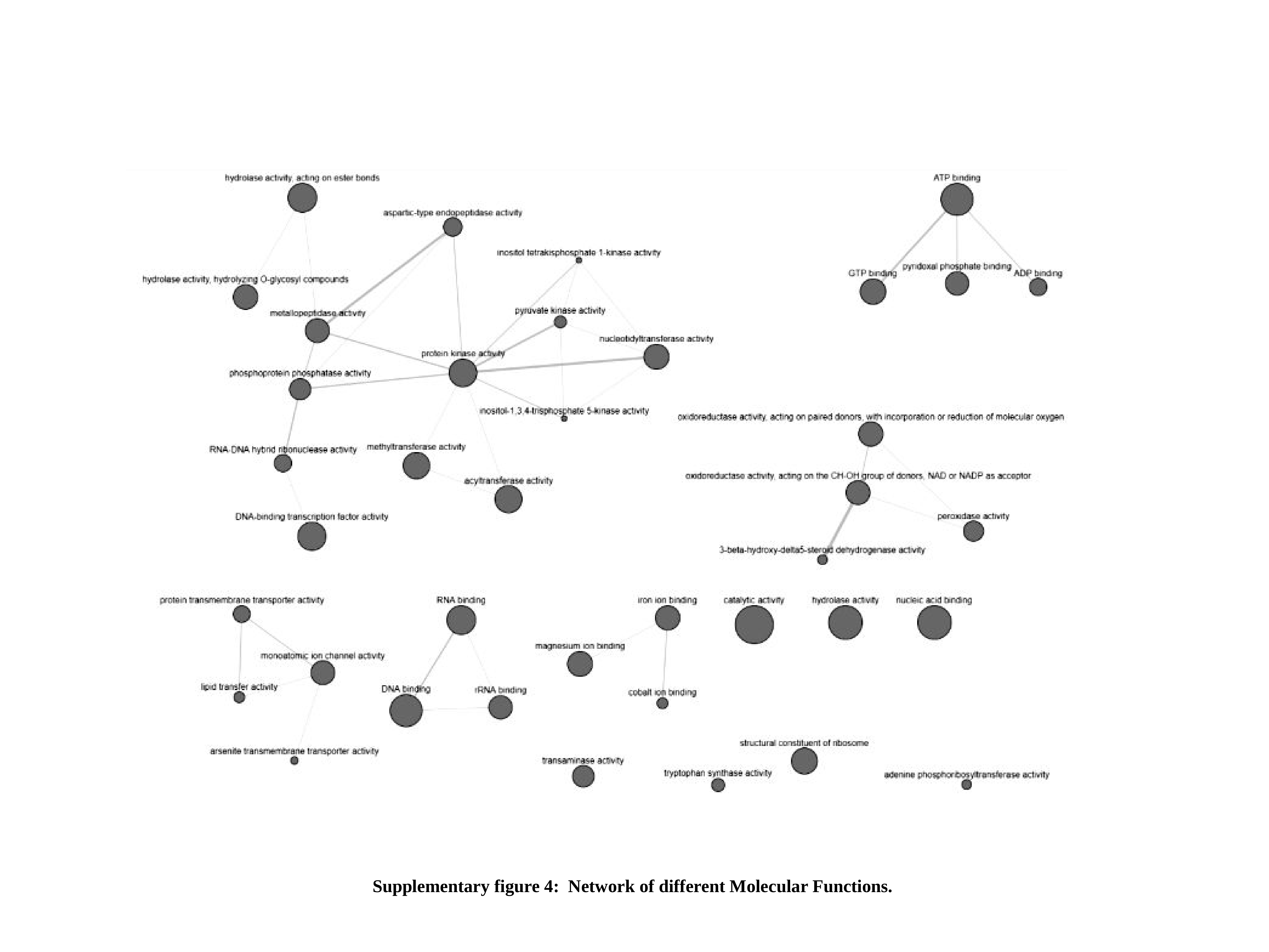

# Supplementary figure 4: Network of different Molecular Functions.
