# Supplementary material for: Genome-wide identification and association analysis of informative SNPs of various nutri-nutraceutical traits in Buckwheat (Fagopyrum spp.)
Source: Front Plant Sci. 2025 Apr 24;16:1559621. doi: 10.3389/fpls.2025.1559621 (PMC12059574; doi:10.3389/fpls.2025.1559621)
Supplement: Supplementary file 1 [file SupplementaryFile1.zip › Supplementary Material/Supplementary Tables 1-3.docx]

**Supplementary Table1: List of Buckwheat germplasm (collected from North Western Himalayas region and from NBPGR, New Delhi)**.

| **S. No.** | **Genotype** | **Species** | **Place of Collection and year of collection/Procured** | **IC/EC Number** | **S. No.** | **Genotype** | **Species** | **Place of Collection and year of collection Procured** | **IC/EC Number** |
| --- | --- | --- | --- | --- | --- | --- | --- | --- | --- |
| 1 | BWZ-1 | *Fagopyrumtartaricum* | Gurez (2018) | - | 30 | BWZ-31 | *Fagopyrumesculentum* | Budnumbal/Kupwara(2018) | IC-0637156 |
| 2 | BWZ-2 | *Fagopyrumesculentum* | Gurez(2018) | - | 31 | BWZ-32 | *Fagopyrumtartaricum* | Sukurbuchan/Leh(2018) | IC-0637176 |
| 3 | BWZ-3 | *Fagopyrumtartaricum* | Gurez(2018) | - | 32 | BWZ-33 | *Fagopyrumtartaricum* | Farona/Kargil(2018) | - |
| 4 | BWZ-4 | *Fagopyrumtartaricum* | Budnambal/Kupwara(2018) | - | 33 | BWZ-34 | *Fagopyrumesculentum* | Takmachik/Leh(2018) | IC-0637157 |
| 5 | BWZ-5 | *Fagopyrumesculentum* | Kargil(2018) | - | 34 | BWZ-35 | *Fagopyrumtartaricum* | Sukurbuchan/Leh(2018) | IC-0637177 |
| 6 | BWZ-6 | *Fagopyrumesculentum* | NBPGR/NewDelhi(2018) | - | 35 | BWZ-36 | *Fagopyrumtartaricum* | Domkhar/Leh(2018) | - |
| 7 | BWZ-7 | *Fagopyrumesculentum* | Kargil(2018) | - | 36 | BWZ-37 | *Fagopyrumtartaricum* | Saliskot/Leh(2018) | - |
| 8 | BWZ-8 | *Fagopyrumtartaricum* | Ladakh(2018(2018)) | - | 37 | BWZ-38 | *Fagopyrumesculentum* | G.M.pora/Kargil(2018) | - |
| 9 | BWZ-9 | *Fagopyrumtartaricum* | NBPGR(2018) | - | 38 | BWZ-39 | *Fagopyrumtartaricum* | Farona/Kargil(2018) | - |
| 10 | BWZ-10 | *Fagopyrumesculentum* | Inshan/Kishtward(2018) | - | 39 | BWZ-40 | *Fagopyrumesculentum* | Sukurbuchan/Leh(2018) | IC-0637158 |
| 11 | BWZ-11 | *Fagopyrumtartaricum* | Wardwan/Kishtward(2018) | IC-0637166 | 40 | BWZ-41 | *Fagopyrumtartaricum* | Sukurbuchan/Leh(2018) | - |
| 12 | BWZ-12 | *Fagopyrumtartaricum* | Kargil(2018) | - | 41 | BWZ-42 | *Fagopyrumtartaricum* | Sukurbuchan/Leh(2018) | - |
| 13 | BWZ-13 | *Fagopyrumtartaricum* | Kargil(2018) | - | 42 | BWZ-43 | *Fagopyrumtartaricum* | Domkhar/Leh(2018) | - |
| 14 | BWZ-14 | *Fagopyrumtartaricum* | Dasi/Gurez(2018) | IC-0637167 | 43 | BWZ-44 | *Fagopyrumesculentum* | Domkhar/Leh(2018) | IC-0637159 |
| 15 | BWZ-15 | *Fagopyrumtartaricum* | Dangan/Gurez(2018) | - | 44 | BWZ-45 | *Fagopyrumesculentum* | Goma/Minji(2018) | IC-0637160 |
| 16 | BWZ-16 | *Fagopyrumtartaricum* | Dangan/Gurez(2018) | - | 45 | BWZ-46 | *Fagopyrumesculentum* | Chachathan/Kargil(2018) | IC-0637161 |
| 17 | BWZ-17 | *Fagopyrumtartaricum* | Dangan/Gurez(2018) | IC-0637168 | 46 | BWZ-47 | *Fagopyrumesculentum* | Farona/Kargil(2018) | IC-0637162 |
| 18 | BWZ-18 | *Fagopyrumtartaricum* | Dangan/Gurez(2018) | - | 47 | BWZ-48 | *Fagopyrumesculentum* | Bhagna/Kargil(2018) | IC-0637163 |
| 19 | BWZ-19 | *Fagopyrumtartaricum* | Dangan/Gurez(2018) | - | 48 | BWZ-49 | *Fagopyrumesculentum* | Dimji/Kishtward(2018) | IC-0637164 |
| 20 | BWZ-20 | *Fagopyrumtartaricum* | Dangan/Gurez(2018) | - | 49 | BWZ-50 | *Fagopyrumesculentum* | Sayia/Kishtward(2018) | IC-0637165 |
| 21 | BWZ-21 | *Fagopyrumtartaricum* | Dangan/Gurez(2018) | IC-0637170 | 50 | BWZ-51 | *Fagopyrumtartaricum* | NBPGR/New Delhi(2018) | IC-13140 |
| 22 | BWZ-22 | *Fagopyrumtartaricum* | Dangan/Gurez(2018) | IC-0637170 | 51 | BWZ-52 | *Fagopyrumtartaricum* | NBPGR/New Delhi(2018) | IC-13143 |
| 23 | BWZ-23 | *Fagopyrumtartaricum* | Dangan/Gurez(2018) | IC-0637171 | 52 | BWZ-53 | *Fagopyrumtartaricum* | NBPGR/New Delhi(2018) | IC-13413 |
| 24 | BWZ-24 | *Fagopyrumtartaricum* | Dangan/Gurez(2018) | IC-0637172 | 53 | BWZ-54 | *Fagopyrumtartaricum* | NBPGR/New Delhi(2018) | IC-14494 |
| 25 | BWZ-25 | *Fagopyrumtartaricum* | Karakbal/Gurez(2018) | IC-0637173 | 54 | BWZ-55 | *Fagopyrumesculentum* | NBPGR/New Delhi(2018) | IC-16555 |
| 26 | BWZ-26 | *Fagopyrumtartaricum* | Karakbal/Gurez(2018) | IC-0637174 | 55 | BWZ-56 | *Fagopyrumtartaricum* | NBPGR/New Delhi(2018) | IC-17370 |
| 27 | BWZ-27 | *Fagopyrumtartaricum* | Dasi/Gurez(2018) | IC-0637175 | 56 | BWZ-57 | *Fagopyrumesculentum* | NBPGR/New Delhi(2018) | IC-17371 |
| 28 | BWZ-28 | *Fagopyrumesculentum* | Surchey/Kargil(2018) | - | 57 | BWZ-58 | *Fagopyrumesculentum* | NBPGR/New Delhi(2018) | IC-17372 |
| 29 | BWZ-29 | *Fagopyrumesculentum* | Saliskot/Kargil(2018) | - | 58 | BWZ-59 | *Fagopyrumesculentum* | NBPGR/New Delhi(2018) | IC-17971 |

| **S. No.** | **Genotype** | **Species** | **Collection/Procured** | **IC/EC Number** | **S. No.** | **Genotype** | **Species** | **Collection/Procured** | **IC/EC Number** |
| --- | --- | --- | --- | --- | --- | --- | --- | --- | --- |
| 59 | BWZ-60 | *Fagopyrumesculentum* | NBPGR/New Delhi(2018) | IC-18040 | 87 | BWZ-90 | *Fagopyrumtartaricum* | NBPGR/New Delhi(2018) | EC-99945 |
| 60 | BWZ-61 | *Fagopyrumtartaricum* | NBPGR/New Delhi(2018) | IC-18751 | 88 | BWZ-91 | *Fagopyrumtartaricum* | NBPGR/New Delhi(2018) | EC-99946 |
| 61 | BWZ-62 | *Fagopyrumtartaricum* | NBPGR/New Delhi(2018) | IC-18757 | 89 | BWZ-92 | *Fagopyrumtartaricum* | NBPGR/New Delhi(2018) | EC-99948 |
| 62 | BWZ-63 | *Fagopyrumesculentum* | NBPGR/New Delhi(2018) | IC-18801 | 90 | BWZ-93 | *Fagopyrumtartaricum* | NBPGR/New Delhi(2018) | EC-104036 |
| 63 | BWZ-64 | *Fagopyrumesculentum* | NBPGR/New Delhi(2018) | IC-18881 | 91 | BWZ-94 | *Fagopyrumtartaricum* | NBPGR/New Delhi(2018) | EC-104037 |
| 64 | BWZ-65 | *Fagopyrumtartaricum* | NBPGR/New Delhi(2018) | IC-18889 | 92 | BWZ-95 | *Fagopyrumesculentum* | NBPGR/New Delhi(2018) | EC-125935 |
| 65 | BWZ-66 | *Fagopyrumtartaricum* | NBPGR/New Delhi(2018) | IC-22426 | 93 | BWZ-97 | *Fagopyrumesculentum* | NBPGR/New Delhi(2018) | EC-125938 |
| 66 | BWZ-67 | *Fagopyrumtartaricum* | NBPGR/New Delhi(2018) | IC-24296 | 94 | BWZ-98 | *Fagopyrumesculentum* | NBPGR/New Delhi(2018) | E-125939 |
| 67 | BWZ-68 | *Fagopyrumtartaricum* | NBPGR/New Delhi(2018) | IC-24298 | 95 | BWZ-99 | *Fagopyrumtartaricum* | NBPGR/New Delhi(2018) | EC-131622 |
| 68 | BWZ-69 | *Fagopyrumtartaricum* | NBPGR/New Delhi(2018) | IC-24299 | 96 | BWZ-100 | *Fagopyrumtartaricum* | NBPGR/New Delhi(2018) | EC-161415-16 |
| 69 | BWZ-70 | *Fagopyrumtartaricum* | NBPGR/New Delhi(2018) | IC-24302 | 97 | BWZ-101 | *Fagopyrumesculentum* | NBPGR/New Delhi(2018) | EC-213685 |
| 70 | BWZ-71 | *Fagopyrumesculentum* | NBPGR/New Delhi(2018) | IC-25744 | 98 | BWZ-102 | *Fagopyrumtartaricum* | NBPGR/New Delhi(2018) | EC-21662 |
| 71 | BWZ-72 | *Fagopyrumtartaricum* | NBPGR/New Delhi(2018) | IC-25999 | 99 | BWZ-104 | *Fagopyrumesculentum* | NBPGR/New Delhi(2018) | EC-216631 |
| 72 | BWZ-73 | *Fagopyrumesculentum* | NBPGR/New Delhi(2018) | IC-26549 | 100 | BWZ-105 | *Fagopyrumesculentum* | NBPGR/New Delhi(2018) | EC-216634 |
| 73 | BWZ-74 | *Fagopyrumesculentum* | NBPGR/New Delhi(2018) | IC-26586 | 101 | BWZ-107 | *Fagopyrumesculentum* | NBPGR/New Delhi(2018) | EC-218734 |
| 74 | BWZ-75 | *Fagopyrumtartaricum* | NBPGR/New Delhi(2018) | IC-26591 | 102 | BWZ-108 | *Fagopyrumesculentum* | NBPGR/New Delhi(2018) | EC-218740 |
| 75 | BWZ-76 | *Fagopyrumtartaricum* | NBPGR/New Delhi(2018) | IC-37277 | 103 | BWZ-109 | *Fagopyrumesculentum* | NBPGR/New Delhi(2018) | EC-218742 |
| 76 | BWZ-77 | *Fagopyrumtartaricum* | NBPGR/New Delhi(2018) | IC-37278 | 104 | BWZ-110 | *Fagopyrumesculentum* | NBPGR/New Delhi(2018) | EC-218784 |
| 77 | BWZ-78 | *Fagopyrumesculentum* | NBPGR/New Delhi(2018) | IC-37281 | 105 | BWM-2 | *Fagopyrumtartaricum* | NBPGR/New Delhi(2018) | IC-24298 |
| 78 | BWZ-80 | *Fagopyrumesculentum* | NBPGR/New Delhi(2018) | IC-37284 | 106 | BWM-9 | *Fagopyrumtartaricum* | NBPGR/New Delhi(2018) | IC-26591 |
| 79 | BWZ-81 | *Fagopyrumtartaricum* | NBPGR/New Delhi(2018) | EC-12537 | 107 | BWM-11 | *Fagopyrumtartaricum* | NBPGR/New Delhi(2018) | IC-107960 |
| 80 | BWZ-82 | *Fagopyrumesculentum* | NBPGR/New Delhi(2018) | EC-18132 | 108 | BWM-12 | *Fagopyrumtartaricum* | NBPGR/New Delhi(2018) | IC-107962 |
| 81 | BWZ-83 | *Fagopyrumtartaricum* | NBPGR/New Delhi(2018) | EC-18182 | 109 | BWM-13 | *Fagopyrumtartaricum* | NBPGR/New Delhi(2018) | IC-107964 |
| 82 | BWZ-84 | *Fagopyrumesculentum* | NBPGR/New Delhi(2018) | EC-18237 | 110 | BWM-14 | *Fagopyrumtartaricum* | NBPGR/New Delhi(2018) | IC-107967 |
| 83 | BWZ-85 | *Fagopyrumtartaricum* | NBPGR/New Delhi(2018) | EC-18629 | 111 | BWM-19 | *Fagopyrumtartaricum* | NBPGR/New Delhi(2018) | IC-107976 |
| 84 | BWZ-86 | *Fagopyrumtartaricum* | NBPGR/New Delhi(2018) | EC-18740 | 112 | BWM-21 | *Fagopyrumtartaricum* | NBPGR/New Delhi(2018) | IC-26549 |
| 85 | BWZ-87 | *Fagopyrumesculentum* | NBPGR/New Delhi(2018) | EC-18781 | 113 | BWM-23 | *Fagopyrumtartaricum* | NBPGR/New Delhi(2018) | IC-107807 |
| 86 | BWZ-89 | *Fagopyrumesculentum* | NBPGR/New Delhi(2018) | EC-18827 | 114 | BWM-25 | *Fagopyrumtartaricum* | NBPGR/New Delhi(2018) | IC-107971 |

| **S. No.** | **Genotype** | **Species** | **Collection/Procured** | **IC/EC Number** | **S. No.** | **Genotype** | **Species** | **Collection/Procured** | **IC/EC Number** |
| --- | --- | --- | --- | --- | --- | --- | --- | --- | --- |
| 115 | BWM-26 | *Fagopyrumtartaricum* | NBPGR/New Delhi(2018) | IC-107972 | 124 | BWM-38 | *Fagopyrumesculentum* | NBPGR/New Delhi(2018) | EC-218940 |
| 116 | BWM-27 | *Fagopyrumtartaricum* | NBPGR/New Delhi(2018) | IC-107982 | 125 | BWM-39 | *Fagopyrumesculentum* | NBPGR/New Delhi(2018) | EC-272442 |
| 117 | BWM-29 | *Fagopyrumtartaricum* | NBPGR/New Delhi(2018) | IC-108501 | 126 | BWM-40 | *Fagopyrumesculentum* | NBPGR/New Delhi(2018) | EC-286379 |
| 118 | BWM-30 | *Fagopyrumtartaricum* | NBPGR/New Delhi(2018) | IC-108505 | 127 | BWM-42 | *Fagopyrumesculentum* | NBPGR/New Delhi(2018) | EC-323723 |
| 119 | BWM-32 | *Fagopyrumtartaricum* | NBPGR/New Delhi(2018) | IC-108509 | 128 | BWM-45 | *Fagopyrumesculentum* | NBPGR/New Delhi(2018) | EC-323731 |
| 120 | BWM-33 | *Fagopyrumtartaricum* | NBPGR/New Delhi(2018) | IC-108511 | 129 | BWM-46 | *Fagopyrumesculentum* | NBPGR/New Delhi(2018) | EC-386667 |
| 121 | BWM-35 | *Fagopyrumesculentum* | NBPGR/New Delhi(2018) | EC-18132 | 130 | BWM-47 | *Fagopyrumesculentum* | NBPGR/New Delhi(2018) | EC-386668 |
| 122 | BWM-36 | *Fagopyrumesculentum* | NBPGR/New Delhi(2018) | EC-18225 | 131 | BWM-48 | *Fagopyrumesculentum* | NBPGR/New Delhi(2018) | EC-386669 |
| 123 | BWM-37 | *Fagopyrumesculentum* | NBPGR/New Delhi(2018) | EC-159498 | 132 | BWM-49 | *Fagopyrumesculentum* | NBPGR/New Delhi(2018) | EC-386671 |

**Supplementary Table 2: Mean values of 132 buckwheat genotypes for Phenol, Flavanoid, Antioxidants, Methionine, Lysine, Protein content, Nitrogen, Iron, Zinc and Ascorbic acid**

| **Genotype** | **Phenol (mg/g)** | **Flavonoid (mg/1000g)** | **Antioxidants (**µg/g**)** | **Methionine(**g/16gN) | **Lysine(**g/16gN) | **Protein content(%)** | **Nitrogen(%)** | **Iron (ppm)** | **Zinc (ppm)** | **Ascorbic acid (µg/g)** |
| --- | --- | --- | --- | --- | --- | --- | --- | --- | --- | --- |
| **BWZ-1** | 3.53±0.011 | 275.92± 0.5 | 20.30 ±0.076 | 1.64±0.10 | 5.98±0.16 | 14.667±0.188 | 2.347±0.030 | 80.5 ± 0.057 | 17.05 ± 0.02 | 0.40 ± 0.041 |
| **BWZ-2** | 2.16±0.011 | 242.03 ±0.96 | 21.19 ±0.026 | 2.47±0.08 | 5.39±0.24 | 16.650±0.236 | 2.664±0.037 | 144.2 ±0.088 | 13.26 ±0.16 | 0.28 ± 0.018 |
| **BWZ-3** | 3.55 ± 0.012 | 275.35 ±0.96 | 20.44 ±0.028 | 1.99±0.07 | 5.72±0.23 | 12.233±0.448 | 1.957±0.072 | 110.3 ±0.088 | 54. 04 ± 0.10 | 0.15 ± 0.028 |
| **BWZ-4** | 3.34 ±0.011 | 305.14 ±0.93 | 20.46 ±0.032 | 1.72±0.10 | 6.42±0.30 | 12.643±0.322 | 2.023±0.052 | 122.43 ±0.120 | 12.78±0.01 | 0.15 ± 0.029 |
| **BWZ-5** | 1.97 ±0.008 | 205.71 ±0.77 | 21.07 ±0.008 | 2.03±0.12 | 5.21±0.17 | 15.987±0.320 | 2.558±0.051 | 96.4 ±0.057 | 51.66±0.06 | 0.20 ± 0.040 |
| **BWZ-6** | 2.02 ±0.005 | 203.59 ±0.76 | 19.38 ±0.063 | 2.40±0.11 | 5.27±0.13 | 12.520±0.289 | 2.003±0.046 | 91.3 ±0.088 | 23.27±0.03 | 0.11 ± 0.021 |
| **BWZ-7** | 2.53 ±0.011 | 208.63 ±0.96 | 21.05 ±0.008 | 3.52±0.07 | 6.68±0.22 | 6.100±0.321 | 0.976±0.051 | 138.43 ±0.088 | 13.37±0.02 | 0.15 ± 0.030 |
| **BWZ-8** | 2.40 ±0.011 | 167.01 ±0.69 | 21.05 ±0.005 | 3.09±0.08 | 6.01±0.15 | 7.117±0.252 | 1.139±0.040 | 89.1 ±0.1 | 15.28±0.03 | 0.09 ± 0.019 |
| **BWZ-9** | 3.47 ±0.012 | 285.48 ±0.98 | 20.47 ±0.041 | 3.40±0.05 | 6.63±0.19 | 8.033±0.318 | 1.285±0.051 | 97.5 ±0.088 | 52.14±0.09 | 0.15 ± 0.030 |
| **BWZ-10** | 1.33 ±0.008 | 167.37 ±0.44 | 21.15 ±0.006 | 3.55±0.11 | 6.33±0.27 | 6.483±0.303 | 1.037±0.049 | 110.6 ±0.057 | 17.75±0.13 | 0.06 ± 0.016 |
| **BWZ-11** | 3.74 ±0.006 | 382.84 ±0.74 | 19.8 ±0.045 | 3.24±0.13 | 6.42±0.17 | 8.583±0.300 | 1.373±0.048 | 151.5 ±0.1 | 19.34±0.03 | 0.16 ± 0.032 |
| **BWZ-12** | 2.62±0.045 | 302.16 ±0.99 | 19.23 ±0.039 | 3.44±0.24 | 5.27±0.16 | 8.887±0.144 | 1.420±0.025 | 149.4 ±0.088 | 15.52±0.07 | 0.16 ± 0.030 |
| **BWZ-13** | 2.53±0.095 | 264.34 ±0.74 | 20.81 ±0.031 | 3.44±0.12 | 5.38±0.16 | 8.033±0.376 | 1.285±0.060 | 80.5 ±0.088 | 17.40±0.23 | 0.12 ± 0.024 |
| **BWZ-14** | 3.76±0.008 | 390.69 ±0.43 | 19.81 ±0.031 | 3.49±0.05 | 6.53±0.10 | 6.673±0.405 | 1.068±0.065 | 148.6 ±0.088 | 40.71± 0.03 | 0.07 ± 0.017 |
| **BWZ-15** | 3.86±0.029 | 424.30 ±0.71 | 18.34 ±0.038 | 3.28±0.12 | 6.63±0.36 | 6.367±0.233 | 1.019±0.037 | 115.5 ±0.088 | 22.78±0.06 | 0.18 ± 0.036 |
| **BWZ-16** | 3.77±0.020 | 416.49 ±0.43 | 19.77 ±0.066 | 3.51±0.07 | 6.46±0.08 | 8.683±0.377 | 1.389±0.060 | 163.1 ±0.133 | 17.56±0.19 | 0.20 ± 0.040 |
| **BWZ-17** | 3.80±0.014 | 383.31 ±0.27 | 19.81 ±0.033 | 3.33±0.05 | 6.44±0.24 | 11.567±0.348 | 1.851±0.056 | 160.4 ±0.088 | 16.49±0.16 | 0.28 ± 0.059 |
| **BWZ-18** | 3.72±0.015 | 390.77 ±0.84 | 20.02 ±0.003 | 3.51±0.10 | 6.52±0.11 | 9.187±0.158 | 1.470±0.025 | 110.4 ±0.057 | 28.18 ±0.40 | 0.19 ± 0.038 |
| **BWZ-19** | 3.79±0.008 | 410.98 ±0.72 | 19.70 ±0.049 | 3.49±0.09 | 6.54±0.19 | 9.020±0.174 | 1.443±0.028 | 104.5±0.057 | 21.12±0.05 | 0.17 ± 0.034 |
| **BWZ-20** | 3.72 ±0.015 | 418.63±0.96 | 20.02 ±0.008 | 2.04±0.08 | 6.30±0.24 | 12.520±0.289 | 2.003±0.046 | 150.3±0.057 | 16.16±0.02 | 0.09 ± 0.019 |
| **BWZ-21** | 3.79± 0.008 | 408.60±0.96 | 17. 48 ±0.100 | 3.13±0.14 | 6.61±0.33 | 11.567±0.348 | 1.851±0.056 | 192.7 ±0.057 | 19.43±0.03 | 0.10 ± 0.019 |
| **BWZ-22** | 3.68±0.026 | 349.68 ±0.57 | 20.06 ±0.003 | 1.52±0.09 | 6.45±0.03 | 7.560±0.295 | 1.210±0.047 | 171.4 ±0.088 | 19.43 ±0.07 | 0.19 ± 0.038 |
| **BWZ-23** | 3.74±0.023 | 442.80 ±0.78 | 19.35 ±0.051 | 2.36±0.07 | 6.40±0.13 | 11.700±0.361 | 1.872±0.058 | 117.6 ±0.120 | 19.16 ±0.03 | 0.42 ± 0.092 |
| **BWZ-24** | 3.76 ±0.008 | 459. 81 ±0.44 | 19.70 ±0.024 | 3.49±0.16 | 5.76±0.18 | 5.810±0.156 | 0.930±0.025 | 166.5±0.115 | 16.33±0.07 | 0.35 ± 0.077 |
| **BWZ-25** | 3.74 ±0.012 | 359.83 ±0.42 | 20.39 ±0.031 | 3.44±0.14 | 6.51±0.25 | 15.457±0.292 | 2.473±0.047 | 137.7±0.057 | 22.03±0.02 | 0.09 ± 0.019 |
| **BWZ-26** | 3.36 ±0.103 | 475.5 ±0.99 | 19.75 ±0.060 | 2.12±0.11 | 6.15±0.87 | 7.450±0.293 | 1.192±0.047 | 148.5±0.115 | 17.72±0.03 | 0.19 ± 0.038 |
| **BWZ-27** | 3.78 ±0.003 | 385.49 ±0.99 | 20.47 ±0.057 | 2.49±0.08 | 5.57±0.17 | 6.367±0.233 | 1.019±0.037 | 154.6±0.057 | 14.25±0.03 | 0.29 ± 0.061 |
| **BWZ-28** | 3.00 ±0.074 | 349.82 ±0.43 | 20.56 ±0.107 | 2.16±0.06 | 5.45±0.30 | 7.933±0.233 | 1.267±0.035 | 128.4 ±0.088 | 21.12±0.06 | 0.10 ± 0.019 |
| **BWZ-29** | 2.39 ±0.066 | 285.48 ±0.98 | 23.84 ±0.050 | 3.43±0.17 | 6.76±0.28 | 6.033±0.260 | 0.919±0.021 | 219.7±0.057 | 17.12±0.05 | 0.10 ± 0.020 |
| **BWZ-31** | 2.47 ±0.032 | 255.94±0.98 | 23.53 ±0.088 | 2.63±0.08 | 6.65±0.12 | 17.307±0.249 | 2.767±0.037 | 122.0 ±0.066 | 15.27 ±0.02 | 0.13 ± 0.025 |
| **BWZ-32** | 3.76±0.040 | 404.95 ±0.98 | 20.59 ±0.090 | 4.48±0.24 | 6.91±0.38 | 16.387±0.357 | 2.620±0.055 | 130.3 ±0.088 | 21.36±0.03 | 0.15 ± 0.028 |
| **BWZ-33** | 3.78 ±0.024 | 290.27 ±0.67 | 20.62 ±0.103 | 4.48±0.14 | 6.46±0.12 | 15.590±0.419 | 2.493±0.066 | 170.5 ±0.088 | 21.23±0.05 | 0.13 ± 0.026 |
| **BWZ-34** | 2.40 ±0.062 | 267.58 ±0.66 | 20.18 ±0.033 | 2.26±0.08 | 6.25±0.09 | 14.437±0.296 | 2.310±0.047 | 150.1±0.1 | 15.00 ±0.01 | 0.16 ± 0.032 |
| **BWZ-35** | 3.23 ±0.075 | 281.82 ±0.88 | 19.68 ±0.064 | 3.31±0.05 | 6.40±0.21 | 9.187±0.158 | 1.470±0.025 | 120.6 ±0.088 | 18.24± 0.18 | 0.10 ± 0.019 |
| **BWZ-36** | 3.79 ±0.07 | 356.26±0.66 | 19.67 ±0.064 | 3.26±0.09 | 6.29±0.15 | 8.583±0.300 | 1.373±0.048 | 124.5±0.115 | 18.11±0.05 | 0.03 ±0.017 |
| **BWZ-37** | 3.77 ±0.025 | 454.92 ±0.99 | 19.78 ±0.086 | 1.46±0.06 | 5.56±0.17 | 8.717±0.404 | 1.395±0.065 | 190.4 ±0.057 | 22.05± 0.03 | 0.05 ±0.016 |
| **BWZ-38** | 2.56 ±0.065 | 292.93 ±0.99 | 20.10 ±0.003 | 4.71±0.14 | 6.73±0.18 | 6.400±0.265 | 1.024±0.042 | 105.5±0.057 | 18.35± 0.03 | 0.14 ±0.028 |
| **BWZ-39** | 3.59 ±0.037 | 337.59 ±0.66 | 20.25 ±0.044 | 3.37±0.14 | 5.26±0.19 | 15.457±0.292 | 2.473±0.047 | 102.5 ±0.088 | 18.15± 0.04 | 0.15 ±0.029 |
| **BWZ-40** | 2.44 ±0.023 | 240.25±0.67 | 20.40 ±0.100 | 2.24±0.06 | 6.34±0.21 | 12.643±0.322 | 2.023±0.052 | 122.1 ±0.133 | 19.65± 0.07 | 0.10 ±0.020 |
| **BWZ-41** | 3.61 ±0.055 | 307.59±0.66 | 19.68 ±0.076 | 2.74±0.06 | 6.76±0.08 | 8.100±0.265 | 1.295±0.043 | 110. 1 ± 0.1 | 22.14± 0.04 | 0.05 ±0.016 |
| **BWZ-42** | 3.82 ±0.046 | 411.83 ±0.88 | 19.68 ±0.084 | 3.34±0.04 | 6.30±0.09 | 9.133±0.260 | 1.461±0.042 | 101.6 ±0.060 | 26.03±0.04 | 0.03 ±0.017 |

| **Genotype** | **Phenol** | **Flavonoid** | **Antioxidants** | **Methionine** | **Lysine** | **Protein content** | **Nitrogen** | **Iron** | **Zinc** | **Ascorbic acid** |
| --- | --- | --- | --- | --- | --- | --- | --- | --- | --- | --- |
| **BWZ-43** | 3.64 ±0.049 | 311.81±0.88 | 19.66 ±0.077 | 2.29±0.05 | 6.37±0.18 | 11.467±0.318 | 1.835±0.051 | 120.7±0.115 | 20.11 ±0.01 | 0. 02 ± 0.017 |
| **BWZ-44** | 2.44 ±0.067 | 250.24 ±0.66 | 20.61 ±0.079 | 3.65±0.09 | 5.59±0.02 | 15.650±0.350 | 2.503±0.055 | 123.6 ±0.088 | 19.81 ±0.03 | 0.05 ±0.016 |
| **BWZ-45** | 2.33 ±0.060 | 234.92 ±0.99 | 20.59 ±.043 | 2.12±0.04 | 5.29±0.33 | 7.553±0.408 | 1.207±0.063 | 112.0 ±.066 | 17.72±0.03 | 0.15 ±0.029 |
| **BWZ-46** | 2.38 ±0.024 | 246.25 ±0.66 | 20.57 ±0.096 | 1.63±0.02 | 5.68±.006 | 16.387±0.357 | 2.620±0.055 | 101.5 ±0.088 | 26.57 ±0.04 | 0.09 ± 0.019 |
| **BWZ-47** | 2.67 ±0.068 | 313.59 ±0.33 | 35.64 ±0.047 | 2.58±0.06 | 5.49±0.17 | 14.660±0.375 | 2.343±0.058 | 115.7 ±0.115 | 23.10±0.05 | 0.18 ±0.035 |
| **BWZ-48** | 2.54 ±0.040 | 248.70 ±0.96 | 34.54 ±0.058 | 2.27±0.03 | 5.46±0.33 | 13.483±0.366 | 2.157±0.058 | 121.8 ±0.057 | 19.58 ±0.04 | 0.02 ±0.018 |
| **BWZ-49** | 2.18 ±0.033 | 216.25 ±0.66 | 35.79 ±0.072 | 3.23±0.05 | 5.38 ±0.30 | 11.633±0.410 | 1.860±0.064 | 132.1 ±0.133 | 23.17 ±0.03 | 0.02 ±0.018 |
| **BWZ-50** | 2.18 ±0.055 | 224.93 ±0.99 | 34.79 ±0.118 | 1.42±0.04 | 5.43± 0.04 | 18.227±0.146 | 2.917±0.023 | 122.4 ±0.088 | 16.27±0.08 | 0.21 ±0.043 |
| **BWZ-51** | 3.41 ±0.014 | 357.59 ±0.66 | 31.32 ±0.038 | 2.94±0.02 | 6.79± 0.24 | 9.377±0.204 | 1.500±0.032 | 72.7 ±0.088 | 21.94±0.03 | 0.39 ±0.085 |
| **BWZ-52** | 3.37 ±0.020 | 305.15 ±0.96 | 30.71 ±0.080 | 2.38±0.05 | 6.32± 0.10 | 12.520±0.289 | 2.003±0.046 | 96.5 ±0.088 | 15.32 ±0.05 | 0.33 ±0.071 |
| **BWZ-53** | 3.53 ±0.014 | 386.25 ±0.67 | 27.05 ±0.010 | 2.09±0.04 | 6.12± 0.10 | 11.567±0.348 | 1.851±0.056 | 157.4±0.115 | 23.25 ±0.16 | 0.13 ±0.026 |
| **BWZ-54** | 3.51 ±0.037 | 271.77 ±0.90 | 31.58 ±0.094 | 3.56±0.08 | 6.44± 0.12 | 7.067±0.233 | 1.130±0.038 | 117.6 ±0.088 | 19.08±0.16 | 0.09 ±0.018 |
| **BWZ-55** | 1.84 ±0.037 | 135.21 ±0.90 | 34.55 ±0.070 | 3.47±0.06 | 6.53± 0.26 | 7.337±0.203 | 1.173±0.032 | 91.5±0.115 | 21.12±0.05 | 0.41 ±0.091 |
| **BWZ-56** | 3.35 ±0.008 | 256.26 ±0.66 | 32.71 ±0.082 | 2.09±0.05 | 6.07± 0.05 | 9.220±0.191 | 1.473±0.028 | 191.6 ±0.088 | 20.05±0.03 | 0.07 ±0.017 |
| **BWZ-57** | 2.29 ±0.070 | 176.26 ±0.66 | 33.49 ±0.039 | 2.69±0.05 | 6.69± 0.16 | 15.550±0.278 | 2.487±0.044 | 166.7±0.115 | 21.45±0.03 | 0.07 ±0.017 |
| **BWZ-58** | 2.32 ±0.057 | 286.26 ±0.65 | 26.59 ±0.055 | 1.87±0.05 | 5.44± 0.39 | 13.540±0.291 | 2.167±0.047 | 103.06±0.066 | 26.10±0.05 | 0.10 ±0.019 |
| **BWZ-59** | 3.41 ±0.014 | 309.82 ±0.44 | 35.06 ±0.020 | 2.64±0.02 | 6.45± 0.34 | 11.933±0.233 | 1.907±0.035 | 84.7 ±0.088 | 16.07±0.03 | 0.24 ±0.050 |
| **BWZ-60** | 3.59 ±0.017 | 241.29 ±0.73 | 27.74 ±0.137 | 2.55±0.02 | 5.48± 0.08 | 13.417±0.300 | 2.147±0.048 | 137.4 ±0.033 | 26.25±0.03 | 0.12 ±0.023 |
| **BWZ-61** | 3.40 ±0.014 | 409.82 ±0.44 | 37. 53 ±0.081 | 1.95±0.03 | 6.49± 0.37 | 12.743±0.387 | 2.037±0.060 | 84.4±0.057 | 12. 66 ± 0.03 | 0.08 ±0.017 |
| **BWZ-62** | 3.45 ±0.017 | 304.92 ±0.99 | 35.72 ±0.065 | 1.09±0.05 | 5.65± 0.34 | 14.067±0.176 | 2.248±0.030 | 77.5±0.115 | 21.05±0.02 | 0.08 ±0.017 |
| **BWZ-63** | 1.91 ±0.008 | 281.84 ±0.87 | 30.56 ±0.098 | 2.26±0.03 | 5.13± 0.51 | 9.643±0.446 | 1.543±0.071 | 140.3 ±0.057 | 23.14±0.03 | 0.03 ±0.017 |

| **Genotype** | **Phenol** | **Flavonoid** | **Antioxidants** | **Methionine** | **Lysine** | **Protein content** | **Nitrogen** | **Iron** | **Zinc** | **Ascorbic acid** |
| --- | --- | --- | --- | --- | --- | --- | --- | --- | --- | --- |
| **BWZ-64** | 1.32 ±0.020 | 177.59 ±0.66 | 20.79 ±0.133 | 4.13±0.16 | 6.57± 0.45 | 6.383±0.246 | 1.020±0.039 | 178.3 ±0.057 | 17.22±0.06 | 0.07 ±0.017 |
| **BWZ-65** | 3.37 ±0.008 | 244.96 ±0.98 | 20.66 ±0.123 | 3.40±0.08 | 6.37± 0.11 | 7.067±0.348 | 1.130±0.055 | 107.0 ±0.066 | 22.12 ±0.04 | 0.09 ±0.019 |
| **BWZ-66** | 3.23 ±0.060 | 348.30 ±0.68 | 20.33 ±0.120 | 3.36±0.06 | 5.31± 0.09 | 8.523±0.355 | 1.363±0.056 | 84.6 ±0.145 | 14.71±0.03 | 0.17 ±0.033 |
| **BWZ-67** | 3.23 ±0.090 | 324.29 ±0.33 | 21.02 ±.002 | 3.55±0.04 | 5.39± 0.59 | 6.383±0.268 | 1.021±0.043 | 162.6 ±0.088 | 15.50±0.03 | 0.11 ±0.021 |
| **BWZ-68** | 3.29±0.040 | 281.82 ±0.88 | 23.65 ±0.073 | 3.27±0.06 | 5.27± 0.15 | 8.683±0.377 | 1.387±0.058 | 81.5 ±0.115 | 19.13 ±0.03 | 0.14 ±0.027 |
| **BWZ-69** | 3.31±0.005 | 284.96 ±0.98 | 23.51 ±.058 | 2.54±0.04 | 5.62± 0.10 | 12.587±0.347 | 2.013±0.055 | 105.7 ±0.115 | 26.08±0.05 | 0.42 ±0.094 |
| **BWZ-70** | 2.32±0.033 | 195.42 ±0.03 | 23.66 ±0.057 | 3.62±0.05 | 6.60± 0.13 | 9.100±0.289 | 1.452±0.046 | 152.3 ±0.088 | 21.22±0.02 | 0.24 ±0.050 |
| **BWZ-71** | 1.78±0.040 | 159.83 ±0.42 | 25.41 ±0.074 | 3.20±0.05 | 5.55± 0.31 | 9.350±0.218 | 1.500±0.035 | 103.0 ±0.066 | 18.29±0.03 | 0. 60 ± 0.138 |
| **BWZ-72** | 2.04±0.011 | 307.53 ±0.61 | 22.48 ±0.103 | 2.61±0.04 | 6.68± .16 | 10.500±0.289 | 1.680±0.046 | 83.7 ±0.115 | 21.14±0.03 | 0.27 ±0.055 |
| **BWZ-73** | 2.82 ±0.026 | 137.53 ±0.60 | 21.52 ±0.045 | 2.54±0.06 | 5.59± 0.13 | 15.400±0.231 | 2.460±0.035 | 152.6 ±0.115 | 20.50±±0.03 | 0.26 ±0.054 |
| **BWZ-74** | 1.84 ±0.017 | 134.28 ±0.65 | 21.05 ±0.009 | 2.07±0.01 | 5.23± 0.26 | 13.640±0.260 | 2.180±0.040 | 166.6 ±0.088 | 17.29 0.09 | 0.32 ±0.068 |
| **BWZ-75** | 3.32 ±0.006 | 297.49 ±0.57 | 22.67 ±0.069 | 2.21±0.04 | 5.48± 0.28 | 13.540±0.291 | 2.167±0.047 | 156.5 ±0.115 | 24.06 ±0.03 | 0.30 ±0.064 |
| **BWZ-76** | 3.33 ±0.008 | 218.64 ±0.96 | 22.35 ±0.041 | 3.16±0.04 | 6.18± 0.13 | 14.470±0.327 | 2.313±0.050 | 152.0 ±0.066 | 17.10 ±0.06 | 0.33 ±0.070 |
| **BWZ-77** | 3.37 ±0.014 | 248.61 ±0.96 | 26.59 ±0.066 | 3.07±0.02 | 5.73± 0.18 | 13.350±0.236 | 2.097±0.012 | 167.5 ±0.120 | 17.44 ±0.13 | 0.26 ±0.055 |
| **BWZ-78** | 0. 54±0.069 | 117.46 ±0.53 | 25.30 ±0.024 | 1.32±0.05 | 6.38± 0.07 | 18.920±0.395 | 3.027±0.064 | 153.5 ±0.088 | 49.49 ±0.23 | 0.28 ±0.058 |
| **BWZ-80** | 1.86 ±0.049 | 154.24 ±0.64 | 22.46 ±0.057 | 1.64±0.03 | 5.59± 0.14 | 7.403±0.264 | 1.183±0.041 | 173.6 ±0.115 | 21.33 ±0.13 | 0.34 ±0.073 |
| **BWZ-81** | 3.30 ±0.008 | 221.70 ±0.94 | 21.67 ±0.053 | 1.72±0.04 | 5.80± 0.09 | 12.743±0.387 | 2.037±0.060 | 163.7±0.115 | 28.27 ±0.09 | 0.29 ±0.062 |
| **BWZ-82** | 3.20 ±0.07 | 230.76 ±0.10 | 26.56 ±0.063 | 3.54±0.04 | 6.28± 0.26 | 16.353±0.324 | 2.617±0.052 | 173.6 ±0.088 | 30.27 ±0.09 | 0.43 ±0.096 |
| **BWZ-83** | 0.000 | 114.74 ±0.09 | 17.77 ±0.115 | 1.14±0.05 | 6.49± 0.30 | 16.477±0.290 | 2.637±0.046 | 83.06 ±0.066 | 20.09 ±0.03 | 0.23 ±0.046 |
| **BWZ-84** | 1.69 ±0.031 | 158.55 ±0.10 | 22.59 ±0.082 | 1.80±0.04 | 5.65± 0.15 | 9.410±0.231 | 1.503±0.035 | 90.3 ±0.057 | 19.95 ±0.03 | 0.49 ±0.110 |
| **BWZ-85** | 3.40 ±0.011 | 195.02 ±0.99 | 21.58 ±0.042 | 1.34±0.08 | 5.30± 0.24 | 14.503±0.360 | 2.320±0.057 | 137.4±0.057 | 18.33 ±0.01 | 0.37 ±0.080 |

| **Genotype** | **Phenol** | **Flavonoid** | **Antioxidants** | **Methionine** | **Lysine** | **Protein content** | **Nitrogen** | **Iron** | **Zinc** | **Ascorbic acid** |
| --- | --- | --- | --- | --- | --- | --- | --- | --- | --- | --- |
| **BWZ-86** | 2.98 ±0.055 | 199.72 ±0.53 | 25.65 ±0.067 | 2.17±0.05 | 6.41± 0.21 | 11.723±0.382 | 1.873±0.059 | 168.5±0.115 | 23.15 ±0.01 | 0.20 ±0.040 |
| **BWZ-87** | 1.89 ±0.034 | 115.05 ±0.98 | 25.61 ±0.145 | 2.60±0.04 | 5.77± 0.19 | 12.517±0.398 | 2.000±0.061 | 167.7 ±0.088 | 26.16 ±0.03 | 0.26 ±0.053 |
| **BWZ-88** | 1.95 ±0.037 | 128.64 ±0.96 | 17.23 ±0.045 | 1.42±0.04 | 5.38± 0.08 | 13.350±0.236 | 2.133±0.035 | 177.6 ±0.145 | 20.31 ±0.06 | 0.15 ±0.017 |
| **BWZ-90** | 1.60 ±0.017 | 65. 08 ±0.97 | 21.38 ±0.029 | 2.26±0.05 | 6.42± 0.19 | 12.653±0.407 | 2.023±0.064 | 169.4 ±0.145 | 18.32± 0.06 | 0.25 ±0.052 |
| **BWZ-91** | 3.35 ±0.017 | 315.01 ± 0.99 | 21.59 ±0.043 | 3.10±0.06 | 6.33± 0.20 | 11.623±0.314 | 1.860±0.050 | 176.1 ±0.133 | 14.02±0.01 | 0.12 ±0.024 |
| **BWZ-92** | 3.27 ±0.020 | 315.55 ±0.68 | 21.64 ±0.083 | 3.03±0.06 | 5.59± 0.16 | 12.450±0.333 | 1.990±0.051 | 101.6 ±0.088 | 23.26 ±0.04 | 0.27 ±0.055 |
| **BWZ-93** | 3.31±0.017 | 321.37 ±0.80 | 21.29 ±0.044 | 2.50±0.08 | 6.52± 0.22 | 13.640±0.375 | 2.180±0.058 | 173.0 ±0.066 | 19.11±0.03 | 0.19 ±0.038 |
| **BWZ-94** | 3.31 ±0.005 | 287.50 ±0.57 | 25.11 ±0.347 | 2.54±0.07 | 6.42± 0.32 | 14.370±0.233 | 2.297±0.035 | 170.5 ±0.088 | 22.59±0.03 | 0.25 ±0.052 |
| **BWZ-95** | 1.78±0.052 | 145.78 ±0.69 | 23.55 ±0.055 | 2.47±0.05 | 5.41± 0.16 | 15.683±0.377 | 2.507±0.058 | 158.0 ±0.066 | 37.47±0.15 | 0.60 ±0.137 |
| **BWZ-97** | 2.20 ±0.020 | 141.25 ±0.31 | 21.65 ±0.070 | 2.03±0.07 | 5.10± 0.08 | 15.100±0.321 | 2.413±0.052 | 173.5 ±0.152 | 19.08 ±0.04 | 0.53 ±0.119 |
| **BWZ-98** | 2.00 ±0.023 | 131.82 ±0.86 | 21.32 ±0.050 | 3.44±0.03 | 5.38±0.09 | 10.307±0.249 | 1.649±0.040 | 161.4 ±0.088 | 20.39±0.03 | 0.45 ±0.100 |
| **BWZ-99** | 2.00 ±0.095 | 194.43 ±0.05 | 20.46 ±0.043 | 1.56±0.05 | 6.58±0.09 | 17.600±0.379 | 2.813±0.058 | 177.1 ±0.133 | 17.06 ±0.03 | 0.26 ±0.016 |
| **BWZ-100** | 3.46 ±0.044 | 281.03 ±0.36 | 19.91 ±.020 | 3.20±0.04 | 6.28±0.18 | 17.603±0.307 | 2.817±0.049 | 174.6 ±0.115 | 24.22 ±0.06 | 0.37 ±0.081 |
| **BWZ-101** | 3.42 ±0.008 | 295.71 ±0.33 | 23.12 ±0.012 | 2.79±0.03 | 5.51±0.24 | 11.567±0.348 | 1.850±0.055 | 165.5 ±0.115 | 22.29±0.09 | 0.31 ±0.065 |
| **BWZ-102** | 5.74 ±0.026 | 351.10 ±0.32 | 21.62 ±0.096 | 3.34±0.04 | 5.37±0.18 | 10.737±0.406 | 1.717±0.064 | 82.4 ±0.120 | 16.10 ±0.01 | 0.36 ±0.079 |
| **BWZ-104** | 2.46 ±0.011 | 265.72 ±0.31 | 20.41 ±0.047 | 2.58±0.01 | 5.56±0.14 | 10.567±0.348 | 1.690±0.055 | 171.1 ±0.1 | 24.78 ±0.06 | 0.41 ±0.091 |
| **BWZ-105** | 3.23 ±0.020 | 210.55 ±0.34 | 23.73 ±0.088 | 1.61±0.03 | 5.43±0.16 | 16.270±0.241 | 2.603±0.038 | 169.6 ±0.088 | 16.18 ±0.07 | 0.31 ±0.065 |
| **BWZ-107** | 3.26 ±0.014 | 180.98 ±0.40 | 22.85 ±0.031 | 2.16±0.03 | 5.28±0.13 | 14.067±0.291 | 2.217±0.073 | 169.1 ±0.1 | 21.15 ±0.05 | 0.32 ±0.069 |
| **BWZ-108** | 3.21 ±0.045 | 176.86 ±0.25 | 23.51 ±0.095 | 2.15±0.04 | 5.37±0.26 | 15.100±0.321 | 2.413±0.052 | 162.3 ±0.088 | 21.56 ±0.06 | 0.29± 0.060 |
| **BWZ-109** | 3.21±0.005 | 220.72 ±0.35 | 22.55 ±0.059 | 3.34±0.04 | 5.44±0.12 | 9.133±0.260 | 1.459±0.040 | 160.4 ±0.088 | 22.14 ±0.05 | 0.30 ±0.063 |
| **BWZ-110** | 2.82±0.005 | 180.87 ±0.50 | 22.70 ±0.091 | 3.58±0.05 | 5.36±0.19 | 8.390±0.231 | 1.340±0.035 | 162.1 ±0.088 | 19.19 ±0.03 | 0.32 ±0.069 |

| **Genotype** | **Phenol** | **Flavonoid** | **Antioxidants** | **Methionine** | **Lysine** | **Protein content** | **Nitrogen** | **Iron** | **Zinc** | **Ascorbic acid** |
| --- | --- | --- | --- | --- | --- | --- | --- | --- | --- | --- |
| **BWM-2** | 5.12±0.005 | 310.79 ±0.40 | 23.42 ±0.072 | 2.09±0.02 | 5.32±0.16 | 10.403±0.304 | 1.660±0.050 | 167.1 ±0.088 | 38.7 ±0.06 | 0.31 ±0.026 |
| **BWM-9** | 4.30 ±0.012 | 180.60 ±0.32 | 22.23 ±0.066 | 1.80±0.04 | 6.48±0.36 | 12.620±0.376 | 2.019±0.060 | 188.0 ±0.066 | 42.2±0.33 | 0.29 ±0.023 |
| **BWM-11** | 5. 85 ± 0.005 | 194.17 ±0.35 | 20.47 ±0.026 | 1.47±0.40 | 5.63±0.20 | 9.583±0.300 | 1.533±0.048 | 121.0 ±0.033 | 35.56 ±0.25 | 0.21 ±0.021 |
| **BWM-12** | 5.73 ±0.008 | 160.83 ±0.30 | 21.36 ±0.088 | 2.64±0.03 | 6.72±0.10 | 10.500±0.289 | 1.680±0.046 | 126.1 ±0.166 | 40.40 ±.06 | 0.32 ±0.018 |
| **BWM-13** | 5.34 ±0.011 | 141.32 ±0.28 | 21.21 ±0.037 | 2.95±0.03 | 6.72±0.22 | 12.810±0.437 | 2.050±0.070 | 122.1 ±0.166 | 44.5±0.10 | 0.10 ±0.016 |
| **BWM-14** | 3.37±0.049 | 232.02 ±0.37 | 21.87±0.038 | 3.64±0.05 | 5.26±0.18 | 11.757±0.408 | 1.881±0.065 | 224.0± 0.066 | 42.2±0.10 | 0.31 ±0.016 |
| **BWM 19** | 3.42 ±0.064 | 212.99 ±0.33 | 22.80±0.028 | 0 | 0 | 13.517±0.398 | 2.163±0.064 | 133.13± 0.032 | 57.700±0.10 | 0.42 ±0.016 |
| **BWM-21** | 2.07 ±0.005 | 170.75±0.33 | 22.75 ±0.087 | 2.95±0.04 | 5.90±0.03 | 10.670±0.353 | 1.707±0.057 | 143.1 ±0.166 | 34.26 ±0.14 | 0.20 ±0.017 |
| **BWM-23** | 2.97±0.005 | 186.06 ±0.34 | 25.21 ±0.076 | 2.59±0.04 | 5.26±0.26 | 14.310±0.199 | 2.290±0.032 | 164.0 ±0.066 | 46.06 ±0.08 | 0.25 ±0.016 |
| **BWM-25** | 2.95±0.011 | 169.12 ±0.37 | 23.68 ±0.058 | 1.26±0.05 | 5.19±0.06 | 8.557±0.387 | 1.369±0.062 | 204.1 ±0.1 | 31.66 ±0.14 | 0.22 ±0.019 |
| **BWM-26** | 2.67±0.008 | 230.74 ±0.33 | 23. 68±0.988 | 1.58±0.05 | 5.53±0.03 | 13.917±0.277 | 2.227±0.044 | 134.0 ±0.033 | 29.3 ±0.15 | 0.32 ±0.016 |
| **BWM-27** | 3.52±0.008 | 213.86 ±0.28 | 23.68±0.043 | 2.36±0.04 | 5.36±0.05 | 11.600±0.379 | 2.189±0.288 | 164.1 ±0.1 | 43.86 ±0.07 | 0.24 ±0.017 |
| **BWM 29** | 2.65±0.073 | 175.78 ±0.33 | 23.63 ±0.036 | 0 | 5.45±0.06 | 12.653±0.407 | 2.025±0.065 | 174.1 ±0.120 | 37.83 ±0.08 | 0.14 ±0.016 |
| **BWM-30** | 1.82±0.005 | 207.63 ±0.35 | 24.06 ±0.007 | 3.44±0.03 | 6.46±0.40 | 8.033±0.260 | 1.285±0.042 | 134.0 ±0.066 | 24.4 ±0.06 | 0.26 ±0.016 |
| **BWM-32** | 2.59±0.008 | 216.36 ±0.35 | 22.53 ±0.0611 | 2.97±0.06 | 6.46±0.40 | 7.660±0.375 | 1.226±0.060 | 133.1 ±0.133 | 33.46 ±0.08 | 0.29 ±0.018 |
| **BWM 33** | 2.53±0.011 | 157.03 ±0.35 | 22.26 ±0.013 | 0 | 0 | 7.067±0.291 | 1.131±0.046 | 163.0 ±0.066 | 27.6 ±0.06 | 0.34 ±0.012 |
| **BWM 35** | 2.30±0.034 | 188.98 ±0.27 | 22.74 ±0.073 | 0 | 0 | 15.953±0.079 | 2.553±0.013 | 114.0 ±0.066 | 47.23 ±0.13 | 0.31 ±0.016 |
| **BWM-36** | 2.92±0.005 | 240.79 ±0.29 | 23.34 ±0.034 | 2.10±0.04 | 5.50±0.39 | 15.617±0.324 | 2.499±0.052 | 116.1 ±0.166 | 18.16 ±0.08 | 0.20 ±0.016 |
| **BWM-37** | 2.73±0.012 | 178.66 ±0.33 | 23.39 ±0.023 | 3.02±0.09 | 5.55±0.38 | 15.490±0.323 | 2.479±0.052 | 133.0 ±0.066 | 32.93 ±0.08 | 0.24 ±0.032 |
| **BWM-38** | 3.07±0.008 | 210.64 ±0.33 | 23.49 ±0.015 | 2.35 ±0.03 | 5.40±0.12 | 14.933±0.291 | 2.389±0.046 | 136.1 ±0.133 | 36.5 ±0.12 | 0.09 ±0.018 |
| **BWM-39** | 1.83±0.005 | 193.89 ±0.39 | 23.49±0.018 | 1.73±0.02 | 5.48±0.24 | 18.123±0.400 | 2.900±0.064 | 233.1 ±0.166 | 45.53 ±0.12 | 0.09 ±0.016 |

| **Genotype** | **Phenol** | **Flavonoid** | **Antioxidants** | **Methionine** | **Lysine** | **Protein content** | **Nitrogen** | **Iron** | **Zinc** | **Ascorbic acid** |
| --- | --- | --- | --- | --- | --- | --- | --- | --- | --- | --- |
| **BWM-40** | 1.88±0.003 | 195.86 ±0.27 | 23.87 ±0.063 | 3.5±0.03 | 5.51±0.06 | 16.150±0.312 | 2.584±0.050 | 162.0 ±0.03 | 24.5 ±0.12 | 0.16 ±0.017 |
| **BWM-42** | 3.52±0.005 | 250.99 ±0.38 | 23.90±0.043 | 1.91±0.01 | 5.46±0.43 | 17.603±0.307 | 2.817±0.049 | 117.1 ±0.1 | 33.63 ±0.12 | 0.25 ±0.016 |
| **BWM-45** | 1.66±0.008 | 211.10 ±0.38 | 23.57±0.019 | 2.52±0.05 | 6.42±0.11 | 11.723±0.382 | 2.207±0.277 | 186.2 ± 0.23 | 44.26±0.10 | 0.26 ±0.017 |
| **BWM-46** | 2.88±0.005 | 241.48 ±0.36 | 22.68±0.015 | 2.50±0.02 | 5.57±0.14 | 13.640±0.375 | 2.183±0.060 | 185. ±0.1 | 35.16 ±0.08 | 0.22 ±0.017 |
| **BWM-47** | 2.422±0.119 | 197.87 ±0.37 | 21.78 ±0.064 | 1.98±0.02 | 5.69±0.25 | 15.457±0.292 | 2.473±0.047 | 168.1 ±0.1 | 27.06 ±0.08 | 0.29 ±0.017 |
| **BWM-48** | 2.45±0.005 | 180.63 ±0.34 | 21.49 ±0.036 | 2.79±0.03 | 5.44±0.30 | 12.417±0.300 | 1.987±0.048 | 175.0 ±0.06 | 30.46 ±0.14 | 0.09 ±0.016 |
| **BWM-49** | 2.81±0.003 | 220.39 ±0.33 | 21.21 ±0.016 | 3.56±0.04 | 6.33±0.20 | 6.383±0.268 | 1.021±0.043 | 129.2 ±0.2 | 45.03 ±0.07 | 0.15 ±0.016 |

**Supplementary Table 3: Genomic variant annotations of Buckwheat**

| **S. No.** | **Chr** | **Location** | **Ref Residue** | **Mutated Residue** | **Location2** | **Type** |
| --- | --- | --- | --- | --- | --- | --- |
|  | Ft1 | 14639543 | C | A | Ft1:14639543 |  |
|  | Ft1 | 14675782 | T | G | Ft1:14675782 |  |
|  | Ft1 | 34715704 | T | C | Ft1:34715704 |  |
|  | Ft1 | 40004476 | G | A | Ft1:40004476 |  |
|  | Ft1 | 47054068 | G | A | Ft1:47054068 |  |
|  | Ft1 | 56206331 | G | A | Ft1:56206331 |  |
|  | Ft1 | 56207465 | G | A | Ft1:56207465 |  |
|  | Ft1 | 63056676 | A | G | Ft1:63056676 |  |
|  | Ft1 | 63056706 | T | C | Ft1:63056706 |  |
|  | Ft1 | 67182755 | G | T | Ft1:67182755 |  |
|  | Ft2 | 7853900 | C | T | Ft2:7853900 |  |
|  | Ft2 | 7855102 | G | A | Ft2:7855102 |  |
|  | Ft2 | 19941896 | T | C | Ft2:19941896 |  |
|  | Ft2 | 42749461 | A | C | Ft2:42749461 |  |
|  | Ft2 | 44458379 | A | G | Ft2:44458379 |  |
|  | Ft2 | 48025684 | G | T | Ft2:48025684 |  |
|  | Ft2 | 50997418 | C | A | Ft2:50997418 |  |
|  | Ft3 | 18606963 | G | A | Ft3:18606963 |  |
|  | Ft3 | 37853900 | T | A | Ft3:37853900 |  |
|  | Ft4 | 18606963 | G | T | Ft4:18606963 |  |
|  | Ft4 | 23778598 | C | T | Ft4:23778598 | Genic |
|  | Ft4 | 37948541 | G | A | Ft4:37948541 |  |
|  | Ft4 | 43826569 | C | T | Ft4:43826569 |  |
|  | Ft4 | 47186890 | G | A | Ft4:47186890 |  |
|  | Ft5 | 7853900 | T | A | Ft5:7853900 |  |
|  | Ft5 | 18606963 | TC | T | Ft5:18606963 |  |
|  | Ft5 | 19139581 | G | T | Ft5:19139581 | Genic |
|  | Ft5 | 19640595 | C | A | Ft5:19640595 |  |
|  | Ft5 | 24057542 | A | C | Ft5:24057542 |  |
|  | Ft5 | 37948541 | A | T | Ft5:37948541 |  |
|  | Ft5 | 49199435 | A | T | Ft5:49199435 |  |
|  | Ft6 | 10161864 | A | T | Ft6:10161864 |  |
|  | Ft6 | 22925002 | G | A | Ft6:22925002 |  |
|  | Ft6 | 26920730 | T | A | Ft6:26920730 |  |
|  | Ft7 | 36559176 | G | A | Ft7:36559176 |  |
|  | Ft7 | 36559176 | GAAT | G | Ft7:36559176 |  |
|  | Ft7 | 40000527 | T | A | Ft7:40000527 |  |
|  | Ft7 | 40000561 | T | C | Ft7:40000561 |  |
|  | Ft7 | 40000586 | T | A | Ft7:40000586 |  |
|  | Ft7 | 49444717 | C | A | Ft7:49444717 |  |
|  | Ft8 | 23398412 | C | G | Ft8:23398412 |  |
|  | Ft8 | 27853900 | A | C | Ft8:27853900 |  |
|  | Ft3 | 17689541 | G | A | Ft3:17689541 | Genic |
|  | Ft1 | 56716710 | G | T | Ft1:56716710 | Intronic |
